# Control of the post-infarct immune microenvironment through biotherapeutic and biomaterial-based approaches

**DOI:** 10.1007/s13346-023-01290-2

**Published:** 2023-02-10

**Authors:** Shreya S. Soni, Arielle M. D’Elia, Christopher B. Rodell

**Affiliations:** School of Biomedical Engineering, Science and Health Systems, Drexel University, Philadelphia, PA 19104 USA

**Keywords:** Inflammatory disease, Heart failure, Immune modulation, Biomaterials, Biotherapeutics

## Abstract

Ischemic heart failure (IHF) is a leading cause of morbidity and mortality worldwide, for which heart transplantation remains the only definitive treatment. IHF manifests from myocardial infarction (MI) that initiates tissue remodeling processes, mediated by mechanical changes in the tissue (loss of contractility, softening of the myocardium) that are interdependent with cellular mechanisms (cardiomyocyte death, inflammatory response). The early remodeling phase is characterized by robust inflammation that is necessary for tissue debridement and the initiation of repair processes. While later transition toward an immunoregenerative function is desirable, functional reorientation from an inflammatory to reparatory environment is often lacking, trapping the heart in a chronically inflamed state that perpetuates cardiomyocyte death, ventricular dilatation, excess fibrosis, and progressive IHF. Therapies can redirect the immune microenvironment, including biotherapeutic and biomaterial-based approaches. In this review, we outline these existing approaches, with a particular focus on the immunomodulatory effects of therapeutics (small molecule drugs, biomolecules, and cell or cell-derived products). Cardioprotective strategies, often focusing on immunosuppression, have shown promise in pre-clinical and clinical trials. However, immunoregenerative therapies are emerging that often benefit from exacerbating early inflammation. Biomaterials can be used to enhance these therapies as a result of their intrinsic immunomodulatory properties, parallel mechanisms of action (e.g., mechanical restraint), or by enabling cell or tissue-targeted delivery. We further discuss translatability and the continued progress of technologies and procedures that contribute to the bench-to-bedside development of these critically needed treatments.

**Graphical Abstract:**

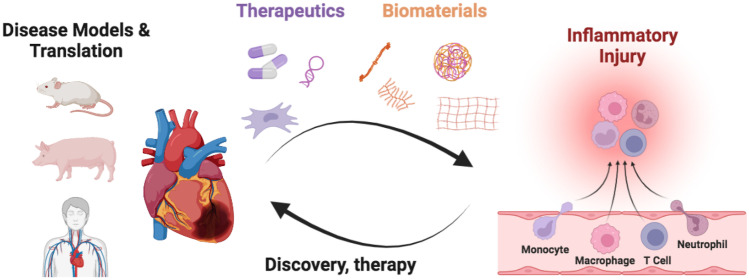

## Introduction

Cardiovascular disease (CVD) remains among the greatest causes of morbidity and mortality, contributing to nearly 30% of deaths worldwide [1]. CVD includes a broad array of conditions, spanning from congenital heart and vascular defects to acquired diseases such as coronary artery disease, myocardial infarction (MI), arrhythmias, and varying presentations of heart failure (HF). In the western world, HF remains the leading cause of death and is expected to afflict nearly 8 million Americans by 2030. Heart disease affects individuals of all socio-demographic backgrounds, with an age-adjusted prevalence varying between whites (11.0%), blacks (9.7%), Hispanics (7.4%), and Asians (6.1%) [2]. The variability among populations has been attributed, in part, to healthcare access, the geographic prevalence of risk factors, and genetic background [3]. Nearly 70% of HF cases are ischemic heart failure (IHF), precipitated by either a partial or complete blockage of blood flow to the myocardium and ensuing left ventricular (LV) remodeling [4, 5]. Atherosclerosis often underlies IHF [6], as continual plaque accumulation, fueled by the accumulation of monocytes (Mo) and macrophages (MF) within the lesion, occludes blood flow to result in myocardial ischemia. Plaque rupture is a primary cause of coronary occlusion and MI.

**Fig. 1:**
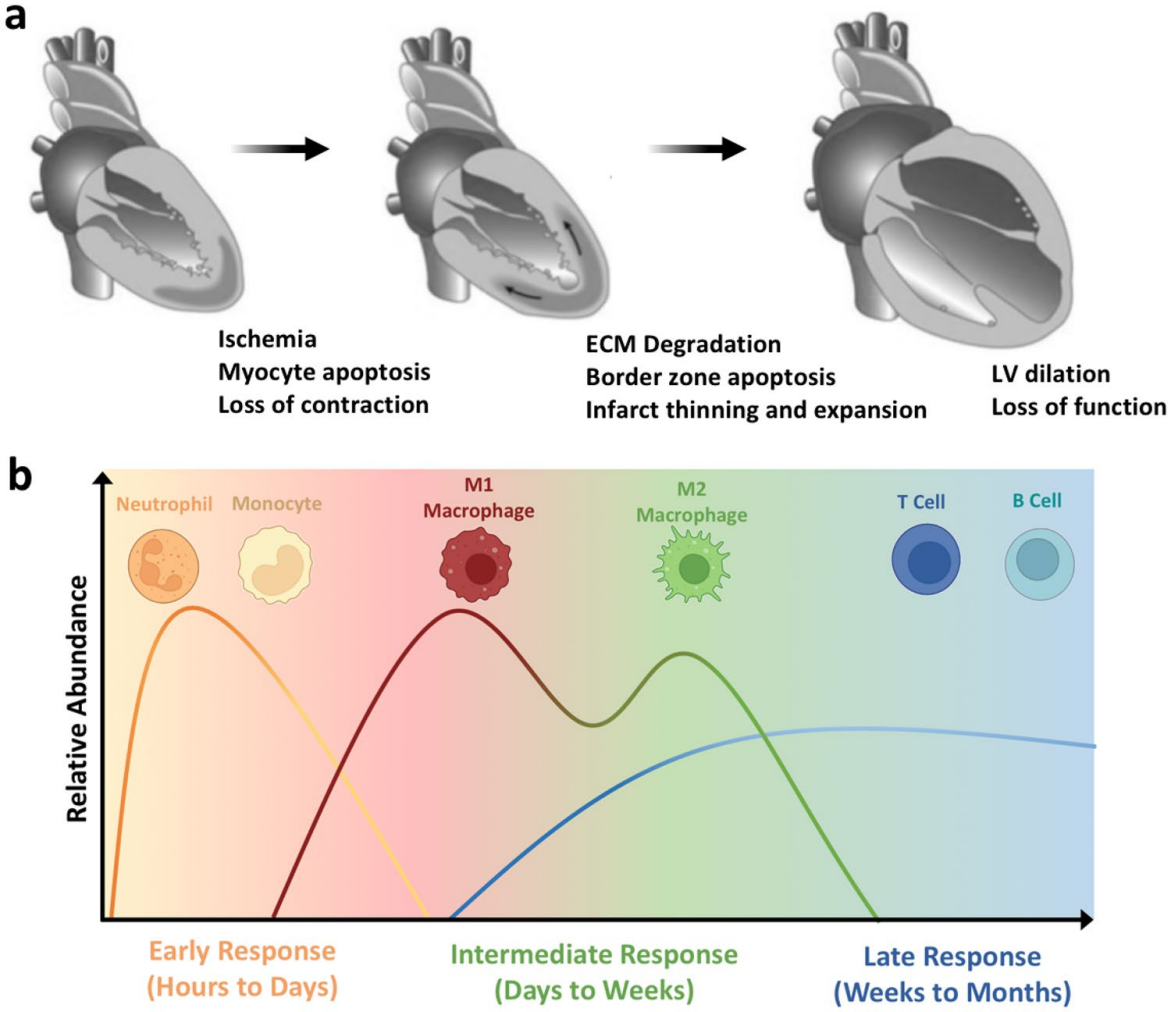
Left ventricular (LV) remodeling and progression of the post-MI inflammatory response.** a** The heart undergoes LV remodeling after MI. Initial ischemia (left) results in cardiomyocyte apoptosis and loss of muscle contraction. Within the following days and weeks, softening of the myocardium by ECM degradation and apoptosis in the border zone result in geometric changes to the heart that include infarct thinning and expansion (middle). Over time, global remodeling is characterized by ventricular dilation, cardiac hypertrophy, and valve dysfunction which result in a loss of heart function that manifest clinically as ischemic heart failure (IHF). Figure adapted from [5]. **b** Following MI, the inflammatory response is incited and can be described in three phases—an early, intermediate, and late response that temporally correlate to the stages of LV remodeling. Innate immune cells (neutrophils, Mo) initially dominate the immune microenvironment, giving way to a wave of inflammatory (M1-like) and later pro-healing (M2-like) MF that exist in a heterogeneous and diverse pool of phenotypes. The inflammatory milieu guides later B and T cell response

The LV remodeling process that occurs after ischemic injury is characterized by maladaptive geometric and functional changes in the heart, which are rooted both in mechanical and inflammatory effects (Fig. 1) [7]. Mediators of the post-MI immune microenvironment are further detailed in Table 1. In the hours and days post-MI, a cascade of ischemia, necrosis, and loss of myocardial contractility result in early expansion of the infarct. This process is paralleled by an early pro-inflammatory response, hallmarked by the rapid recruitment of innate immune cells (neutrophils, Mo, and MF) that begins within minutes post-MI and persists for greater than a week. This robust cellular infiltrate is required for tissue debridement and to initiate repair functions [8]. However, the molecular signals responsible for innate immune cell infiltration are largely pro-inflammatory chemokines (e.g., IP-10, MCP-1), damage-associated molecular patterns (DAMPs, including cell and extracellular matrix (ECM) debris, HMGB1, etc.), and neutrophil degranulation itself [9–12]. Corresponding cell-surface receptors detect these signals to drive homing to the injury site, also initiating pro-inflammatory signaling cascades that produce cytokines and chemokines, further promoting leukocyte recruitment to the site of injury [13].

**Table 1:** Prevalent cell types and signaling markers with their related function in the immune response post-MI

**Components**	**Functions**
**Immune cells**	
Neutrophils	First responder, rapidly migrate to infarct [50, 51] Produce ROS, pro-inflammatory cytokines (IL-1β, IL-6, TNF-α), and MMPs [50, 51] Degranulation and chemokine production promote further leukocyte infiltration [52] Apoptotic neutrophil phagocytosis promotes anti-inflammatory macrophage activation
Monocytes and macrophages	Monocytes traffic to the infarct largely by CCL2-mediated chemotaxis from the spleen and emergency hematopoiesis [24], differentiate into macrophages [6] Debride the tissue via proteolysis [53] and clear cellular debris [54] Pro-inflammatory cytokine production induces cardiomyocyte hypertrophy or apoptosis [50, 51] Later promote repair, including by stimulation of ECM production and angiogenesis [53, 55]
T cell	
Regulatory	Terminate the pro-inflammatory phase [56] Stimulate fibroblasts [57]
Memory effector	Autoimmune reaction against the myocardium [38]
B cells	Autoantibody production [33]
**Signaling markers**	
Pro-inflammatory cytokines (IL-1, TNF-α, IL-6, INF-γ, etc.)	Initiate innate and adaptive response Promote further recruitment, proliferation, and activation [58, 59]
Anti-inflammatory cytokines (VEGF, TGF-β, IL-10 [55], IL-4, IL-13 [60], etc.)	Transition macrophages from M1-like to M2-like Suppress infiltration of inflammatory cells [57, 60]
Chemokines (CCL2, CCL5, CXCL1 [61], etc.) and chemotactic cues (selectins)	Promote chemotaxis of both immune and non-immune cells [61]

Within the infarct, Mo rapidly differentiate into MF that persist for weeks, expanding the local population by greater than tenfold [14–16]. In this intermediate phase, MF are pleiotropic regulators of the immune microenvironment and mediators of the tissue remodeling process that exhibit a wide spectrum of functional phenotypes, having complementary or even opposing functions. Inflammatory MF are often canonically denoted as conventional (M1-like) cells, regarded as tissue damaging. In contrast, alternatively activated (M2-like) cells are considered a tissue-reparatory phenotype [17]. Post-MI, initial MF populations are predominantly M1-like, contributing substantially to tissue debridement and ECM breakdown [18]. They also produce abundant pro-inflammatory cytokines (IL-1, IL-6, and TNF-α) that induce cardiomyocyte hypertrophy or apoptosis, recruit additional cell populations that support chronic inflammation, and are clinical predictors of IHF mortality [19]. Resulting cytokine-induced cardiomyocyte death and breakdown of ECM by overexpression of matrix metalloproteinase (MMP) significantly contribute to infarct thinning and expansion. In later periods post-MI, typically around 1 week in mice, the emergence of M2-like MF coincides with reparatory signals (e.g., PDGF, IL-10, and TGF-β) essential for angiogenesis, cell viability, and collagen production, respectively [20, 21]. MF therefore contribute substantially to LV remodeling and IHF, but is also critical for later tissue repair due to the opposing function of M1- and M2-like phenotypes. As such, modulation of Mo/MF populations and phenotype has been widely investigated [22]. Owing to their potential reparatory roles, MF depletion impairs healing and worsens outcome [23]. In contrast, reducing the number of infiltrating Mo, such as by CCR2 blockade, reduces infarct size and supports post-MI recovery [24]. This is likely due to CCR2-dependent recruitment of inflammatory Mo subpopulations, while non-classical Mo traffic by alternative means [25].

In the following weeks and months, the transition towards an anti-inflammatory and reparative phase to promote wound healing and scar formation is preferable. However, impediments to this transition often force the myocardium into a chronically inflamed state wherein establishment of chronic inflammation and adaptive immune response play a critical role in the remodeling process. As discussed, activated Mo/MF in the infarct mediate para-inflammation (continued leukocyte recruitment) that drives the formation of a chronic inflammatory milieu [26]. Adaptive immune responses, on the other hand, are relatively specific and mediated predominantly by B and T cells [18, 27–29]. Antigen-presenting cells (dendritic cells (DCs)) and to a lesser extent Mo/MF [30] and potentially neutrophils [31, 32] serve as a critical bridge between the innate and adaptive immune response. B cells are derived from the bone marrow and mature into immunoglobin-secreting plasma cells or memory B cells after encountering an antigen epitope. Evidence is emerging that failures of self-tolerance immune checkpoints can result in auto-antibody production, perpetuating disease progression [33]. In contrast, T cells originate from the thymus and differentiate into effector or memory cells, essential to tissue homeostasis and immune memory. In response to environmental stimuli (cytokine signatures) and presented signals (immune checkpoints, antigens), T cells enact a multitude of inflammatory or anti-inflammation cell programs that impact CVD development and progression [34–36]. Regulatory T cells, though low in numbers following MI, abate the inflammatory response through production of anti-inflammatory cytokines [37]. In contrast, development of an adaptive immune response through memory T cells has been shown to coordinate an autoimmune reaction against the myocardium [38]. For a more thorough discussion of adaptive immune response in HF, the reader is referred to recent reviews on this topic [39, 40].

In sum, inflammatory tissue injury underlies LV remodeling and functional declines that manifest as IHF. The process is fundamentally rooted in dysregulation of both the innate and adaptive immune responses that interdependently contribute to LV remodeling, as characterized by cardiomyocyte apoptosis, ventricular dilatation, and myocardial fibrosis that negatively impact heart function [41–45]. Identifying key regulators of these processes is therefore of great importance toward restoring homeostasis and promoting natural injury resolution. To control the post-MI immune microenvironment, therapies have broadly employed the use of cell and bioactive molecule delivery. Cell-based strategies frequently aim to repair or replace the affected tissue. Stem cell therapies, including embryonic (ESC), mesenchymal (MSC), induced pluripotent (iPSC), and others, have been widely explored both as functional tissue replacements and for their immunomodulatory effects [46–48]. However, the delivery of these cells alone is hindered by low cell retention and survival rates, contributing to poor therapeutic efficacy [49] and motivating the use of biomaterial delivery vehicles. Pharmacological approaches include the systemic or local delivery of exogenous cytokines, chemokines, and small molecule drugs. These methods often seek to modulate the hyperinflammatory post-MI environment as a means of cardioprotection or to enhance the body’s inherent tissue repair capacity. Such pro-regenerative strategies have shown some recent success, particularly in the use of microRNA and small interference RNA (siRNA) to promote cardiac cell regeneration [46]. The systemic administration of anti-inflammatory drugs, however, often results in chronic immunosuppression and an elevated risk of infection. Biomaterial-based drug delivery systems enable cell- and tissue-targeting strategies to overcome these challenges, while also concentrating therapeutic concentrations at the site of action. Therefore, there is a critical need for effective delivery strategies and sustained release approaches that can instruct the injury resolution process, either by prophylactically intercepting disease progression, reorienting the hyperinflammatory milieu towards a reparatory state, or reversing the deleterious chronic and adaptive immune response.

## Experimental models and assessment of inflammatory pathophysiology

**Fig. 2:**
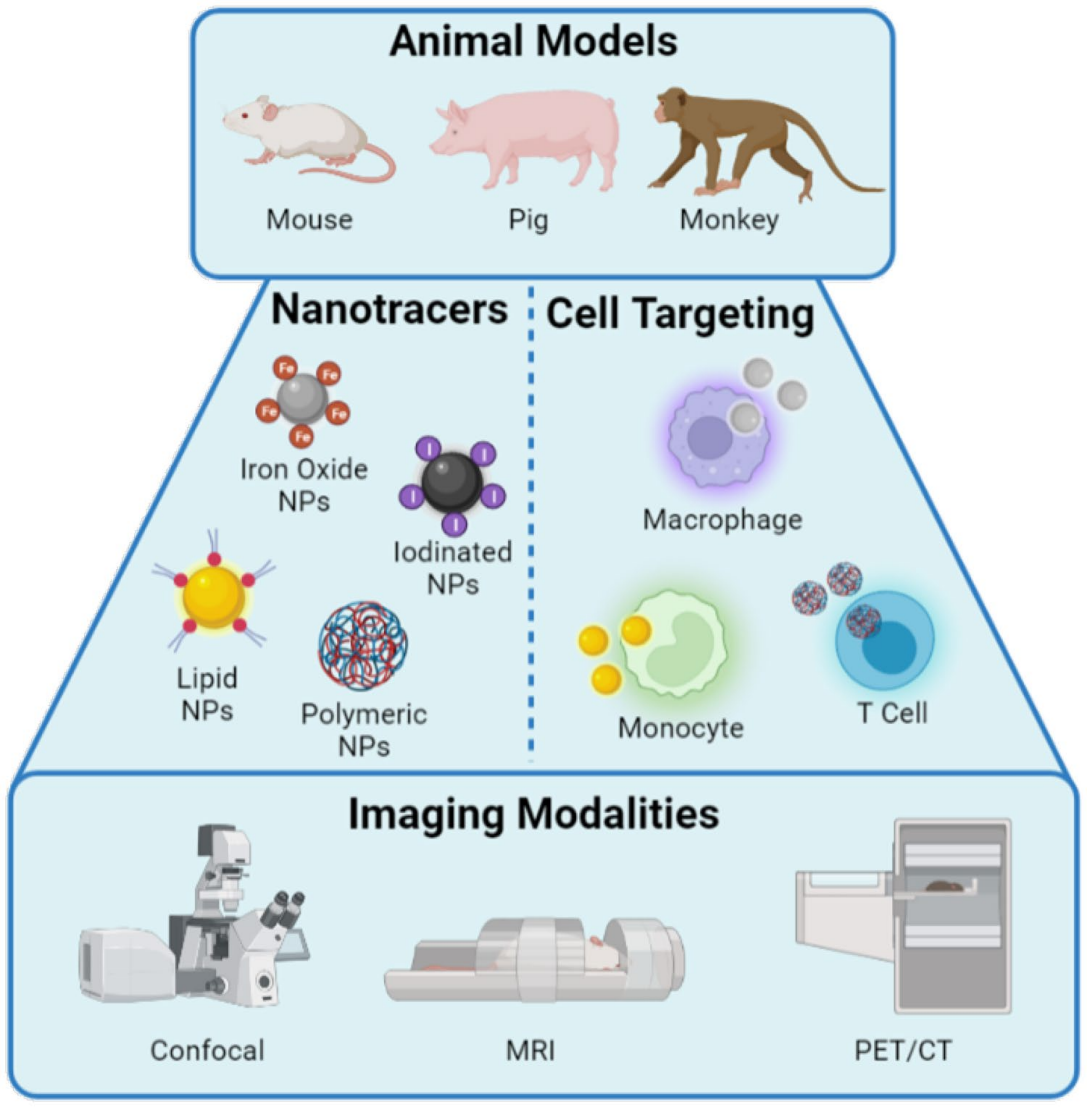
Toolbox of models and methods of image-based assessment. Developing an understanding of IHF etiology and developing therapeutics to treat newly revealed targets requires appropriate selection and pairing of animal models with methods of assessment. Imaging-based assessments are frequently aided by imaging probes or nanotracers, designed to label specific immune cell subsets

Owing to the multifaceted mechanisms of post-MI remodeling processes, the study of both remodeling events and therapeutic strategies to intercept them require investigation in complex environments. These experimental models should recapitulate necessary aspects of the native injury environment that include, for example, complex cell and matrix composition, signaling pathways, and mechanical forces that underlie disease progression. These contributing factors to the pathophysiology evolve over time through a dynamic discourse, which continues to be studied to better understand disease progression and reveal new targets for intervention. Most often, animal models of disease may best recapitulate these processes, as they afford an intact biological system that is a platform for studying the evolution of disease from initial injury to eventual LV remodeling. These studies frequently benefit from advanced imaging approaches, many of which are likewise applicable for diagnosis and as biomarkers for disease stratification (Fig. 2). Here, we provide a brief overview of pertinent methodologies and techniques to delineate the impact of inflammation on IHF.

### Infarct models

In conducting translational research, it is important to choose models with significant prognostic power. In some cases, reductionist approaches are highly applicable to model aspects of tissue injury for this purpose. Microphysiological systems (MPSs) continue to emerge as a powerful means of generating in vitro models for understanding tissue development and screening experimental therapeutics. Construct design requires careful consideration of chemical, physical, and biological cues included—especially for modeling of mechanically actuating and immunological systems [62, 63]. Three-dimensional (3D) printing and other fabrication techniques using immunocompatible materials, such as collagen, gelatin, and chitosan, have shown great promise in the development of artificial heart systems [64]. For example, Boudou and colleagues engineered cardiac microtissues using microelectromechanical systems to facilitate fundamental understanding of cardiovascular biology, develop model systems in vitro, and potentially replace damaged myocardial tissue in vivo. Varying the mechanical stiffness of the collagen cell matrix increased cardiomyocyte contractility, and electrical stimulation and auxotonic load improved cell alignment and force generation, impacting maturation, structure, and function of myocardial tissue [65]. Jackman et al. similarly employed a cell-forward approach to engineer cardiobundles. This system created 3D cylindrical tissue from rat cardiomyocytes or human stem cell-derived cardiomyocytes. Cardiobundles were able to match contractile force, conduction velocity of action potentials, and cardiomyocyte size to mimic those of adult rat tissue [66]. These and related approaches to in vitro cardiac tissue engineering integrate biochemical, biophysical, and electromechanical stimuli to develop physiologically relevant systems for basic discovery and therapeutic benefit [67–69]. While cardiac MPSs confer the potential to study specific aspects of immune cell function in an appropriate dynamic environment, such as by incorporation of tissue resident MF, these applications have yet to be widely explored [70]. Such models are, however, emerging as a means of understanding and screening for cardiotoxicity in the post-MI environment [71].

While benchtop models of disease are suitable for some purposes, a full model for MI should resemble that of the human disease in terms of etiology and pathophysiology. For in-depth evaluation of tissue remodeling processes and therapeutic efficacy, animal models are often most appropriate. For extensive discussion of relevant animal models of MI and appropriate species selection, the reader is referred to prior reviews [72, 73] and expert guidance by Merry Lindsey and colleagues [74]. Small animal models include mice, rats, guinea pigs, and rabbits, which are cost effective, as well as easy to handle and maintain [75]. However, small animals often significantly deviate from human anatomy which may hinder translational potential [76]. Small animal models are nonetheless critical in the research setting to improve our understanding of disease progression and are an invaluable first line of study to evaluate novel treatment strategies. The first model for ischemic injury was established in a Wistar rat using permanent coronary artery ligation by Pfeffer and colleagues [77], and similar models have since been developed in mice and other species [78]. These methods have been adapted to include ischemia–reperfusion (IR) injury, in which blood flow is temporarily occluded to induce ischemic injury and subsequently unblocked to reinstate blood flow [79]. Recent perspectives discuss the clinical relevance of such permanent occlusion versus IR models [80], highlighting that IR recapitulates best clinical standards of care and may best address cardioprotective therapies. On the other hand, permanent occlusion may better replicate clinical pathophysiology of inflammation-driven LV remodeling and is applicable to examination of wound healing, scar formation, and IHF progression.

Utilizing mouse models for ischemic injury provides other added benefits, such as the potential to understand and characterize pathways at the molecular level. Many genetically engineered mice (GEMs) are available for purchase and can be used to systematically and mechanistically understand mechanisms of IHF [81]. Cell-type specific depletion, such as diphtheria toxin receptor (DTR) mouse models, are readily available and can elucidate the role of discrete immune cell populations by simple administration of the toxin. Conditional transgenic DTR models include FOXP3-DTR for T cell depletion [82], Ly6G-DTR for neutrophil depletion [83], and CD11b-DTR for MF depletion [18, 84], among others [85]. These GEMs are a critical tool to manipulate the immune microenvironment for basic discovery. For example, CD169-DTR and CCL2-DTR mice have been used to selectively deplete tissue resident and inflammatory MF populations, which resulted in divergent effects on further Mo recruitment and LV function [86]. By DTR depletion of CX3CR1 cells (cardiac tissue resident MF) and detailed fate mapping and parabiosis studies, the Epelman group has also revealed a cardioprotective role of resident MF that is not redundant with Mo differentiation in the tissue [87]. Similar to limitations involved with other transgenic animal approaches, DTR mouse models rely on accurate choice of cell-specific promoters and the assumption that cells are defined by single promoter activities. This becomes problematic, especially between closely related immune cells such as MF and Mo [85]. As a result, population depletions are often incomplete, tissue-dependent, and temporary. In addition, repeated diphtheria toxin treatments can result in off-target cell effects, sickening or killing the animal [88, 89]. Although DTR transgenic mice and other GEMs [90] permit a greater biological understanding and develop effective therapies, reasonable caution is warranted with their use.

The purchase or generation of genetically modified mouse models for application-specific purposes is also used in practice. Many studies have made use of genetically modified apolipoprotein E-deficient (ApoE^−/−^) mice to study CVD, because they readily reproduce critical aspects of atherosclerosis, the buildup of arterial plaque that is a leading cause of MI, stroke, or angina [91]. Other application-specific models include mice lacking the prostaglandin E2 receptor 4, which has been shown to stimulate cardiomyocyte hypertrophy. After coronary artery ligation to induce MI, the knockout mice showed decreases in hypertrophy, fibrosis, and activation of Stat3, a prominent pro-inflammatory pathway used in T cell maturation relative to wild type [92]. In another example, angiotensin II type IA receptor knockout mice showed decreased levels of TGF-β and fibrosis, which reduced LV remodeling and increased mouse survival [93]. Trib1^−/−^ knockout mice, effectively M2-like MF depleted, experienced more frequent cardiac rupture due to reduced collagen fibril formation in the myocardium. However, administration of M2-like MF and exogenous anti-inflammatory cytokines, like IL-4, restored function of the heart, highlighting the imperative role that M2-like MF play in infarct repair [94]. In sum, small animal models are relatively inexpensive to investigate and GEMs, in particular, greatly enhance the ability to mechanistically understand disease progression and modes of therapeutic action, rendering them critical investigational tools in the field.

Large animal models often confer a higher degree of experimental reliability and biological relevance, as the anatomy and timeline of disease progression is more closely aligned with that of humans. Sheep, pigs, dogs, and baboons are commonly employed large animal models of MI. Details of these models and their common limitations have been previously reviewed elsewhere [95, 96]. Notably, these models do face a degree of logistical challenges, including relatively high costs, as well as greater demands for maintenance and care in laboratory settings [97, 98]. From an ethical perspective, the use of larger animals in scientific research has faced societal criticism, contributing to necessary regulatory oversight [98, 99]. Despite these challenges, larger models of disease are often highly desirable because they possess greater similarities to human anatomy. For example, porcine models closely parallel the coronary vasculature, collateral circulation, and metabolic activity of the human heart, making them an often-preferred model of vascular diseases and intervention [100–102]. Sheep and humans likewise share similar cardiac kinetics and healing patterns following myocardial injury [103, 104]. Non-human primates are most closely related to humans due to their genetic homology. They share significant physiological, metabolic, and biochemical similarities, making them the best model for human disease and intervention [105, 106]. These large animal models are well suited to clinical imaging modalities, such as magnetic resonance imaging (MRI) and computed tomography (CT), and are preferred models for the development of medical devices, such as stents and pacemakers [107]. Regarding the translation of immunotherapeutics, specifically, van Hout et al. have performed a meta-analysis of pre-clinical large animal models treated with anti-inflammatory compounds that have failed to translate to successful clinical trials [108]. Treatments generally led to a reduction in infarct size, supporting the concept of anti-inflammatory therapies. However, the association of these effects with timing, sex, and other experimental variables suggests disparities between pre-clinical and clinical study design that underly translational failures. Specifically, the analysis highlighted that the effect size was greatest when therapeutic intervention occurred early (within 4–8 h post-MI) and when studied only in the male sex. Additionally, procedural mortality was increased when the investigators were blinded to the treatment groups. While both pre-clinical and clinical studies may both blind the investigators, such early intervention is not always clinically feasible and studies should investigate the effect of timing to better understand the effective treatment window. Moreover, pre-clinical investigations should better reflect the population and patient demographics, particularly with regard to sex as a biological variable [109, 110] but also in considering the ancestry of cells such as those used for in vitro studies [111]. As discussed in recent reviews, the interdependence of age and sex is a critical factor in the sexual dimorphism of HF cause, disease progression, and response to treatment [112, 113]. This is particularly pertinent to the study of immunotherapeutics, where response may be influenced either by immunosenescence (a decrease in circulating immune cells and disrupted cytokine response) or inflammaging that is characterized by low-grade chronic inflammation. Considering these factors in experimental design will increase the translational value of research as a whole.

### Imaging techniques

An initial step toward effective therapy includes understanding of disease progression, and a plethora of techniques are available for probing orientation of the immune environment post-MI and assessing functional outcomes. With regard to assessment of LV remodeling, biomedical imaging techniques are widely used in experimental models of CVD and have been recently reviewed elsewhere [114, 115]. These techniques include structural, functional, and biochemical readouts by a variety of techniques that include angiography, echocardiography, MRI, positron emission tomography (PET), CT, and fluorescence imaging. Each of these is a part of the toolbox of techniques available to understand disease progression and therapeutic outcomes (Fig. 2). Through these methods, longitudinal assessment of tissue-scale remodeling (e.g., geometry, tissue microstructure) and also cellular processes (e.g., metabolism, enzymatic activity) is made possible, often paralleled by the development and use of molecular imaging probes [116–120]. However, spatiotemporal insights into the behavior of specific immune cell subsets are notoriously difficult to gain from such whole-body imaging or conventional analysis immunological evaluations (flow cytometry, histology).

A better appreciation of the complex relationship between immune cell subsets is often made possible through cell imaging, including intravital microscopy [121]. Direct imaging of cellular processes in disease states can provide fundamental information about cell homing migration, and interactions that are otherwise inaccessible. An array of suitable fluorescent reporter mice and labeling techniques are available [121, 122] and have been used to provide fundamental insights into cardioimmunology both in the healthy and injured heart. For example, Hulsmans and colleagues used a CX3CR1^GFP/+^ MF reporter mouse to quantify MF abundance in the atrioventricular node and left ventricle, ultimately revealing that MF directly contribute to electrical conduction in the heart via connexin-43-containing gap junctions [123]. Regulatory T cell (Treg) trafficking has been examined in the infarcted myocardium using FoxP3^EGFP^ reporter mice, where Treg depletion increased myocardial dilation, upregulated the expression of CCL2, and accelerated MF infiltration. Treg-targeted therapies, owing to their anti-inflammatory properties, could be a promising method for attenuating post-infarct remodeling [124]. The continued use of single cell imaging is well warranted, and continued advancements, such as intravital microscopy in the beating heart [125], will continue to reveal new targets for immune modulation and are likely to become a fundamental tool for assessment of experimental therapeutics.

In addition to the visualization of immune cell subsets at the cell level, gross evaluation of cellular abundance is also useful as a prognostic and diagnostic biomarker of disease [126, 127]. Biomaterial-based contrast agents and molecular probes to assess immune cell populations and their behavior at the tissue scale are in clinical use and continue to be further developed. MRI is a non-invasive imaging tool, widely used due to its minimal radiation exposure and ready use to detect metallic, paramagnetic, and discrete chemical signatures [128]. Superparamagnetic iron oxide (SPIO) and ultrasmall super paramagnetic iron oxide (USPIO) nanoparticles were developed as negative contrast agents for MRI. These nanoparticles are readily uptaken by MF, which make them advantageous for cell mapping in atherosclerotic plaques, infarcted tissues, and solid tumors. Monocrystalline iron oxide nanoparticle-47 (MION-47) and FDA-approved ferumoxytol are similarly able to detect infiltrating MF in atherosclerosis and the infarcted myocardium [129, 130].

Although angiography and CT are universally used for imaging the coronary artery, specifically detecting MF using CT becomes difficult because high concentrations of absorbent biomaterials are required for the X-ray. However, Hyafil and colleagues developed an iodinated nanoparticulate contrast agent, N1177, that could be uptaken in atherosclerotic MF and subsequently imaged with CT in rabbits to determine MF accumulation in the tissue [131]. Additionally, Cormode et al. characterized MF accumulation in atherosclerotic plaque using their developed gold high-density lipoprotein contrast agent for CT in ApoE^−/−^ mice [132].

**Fig. 3:**
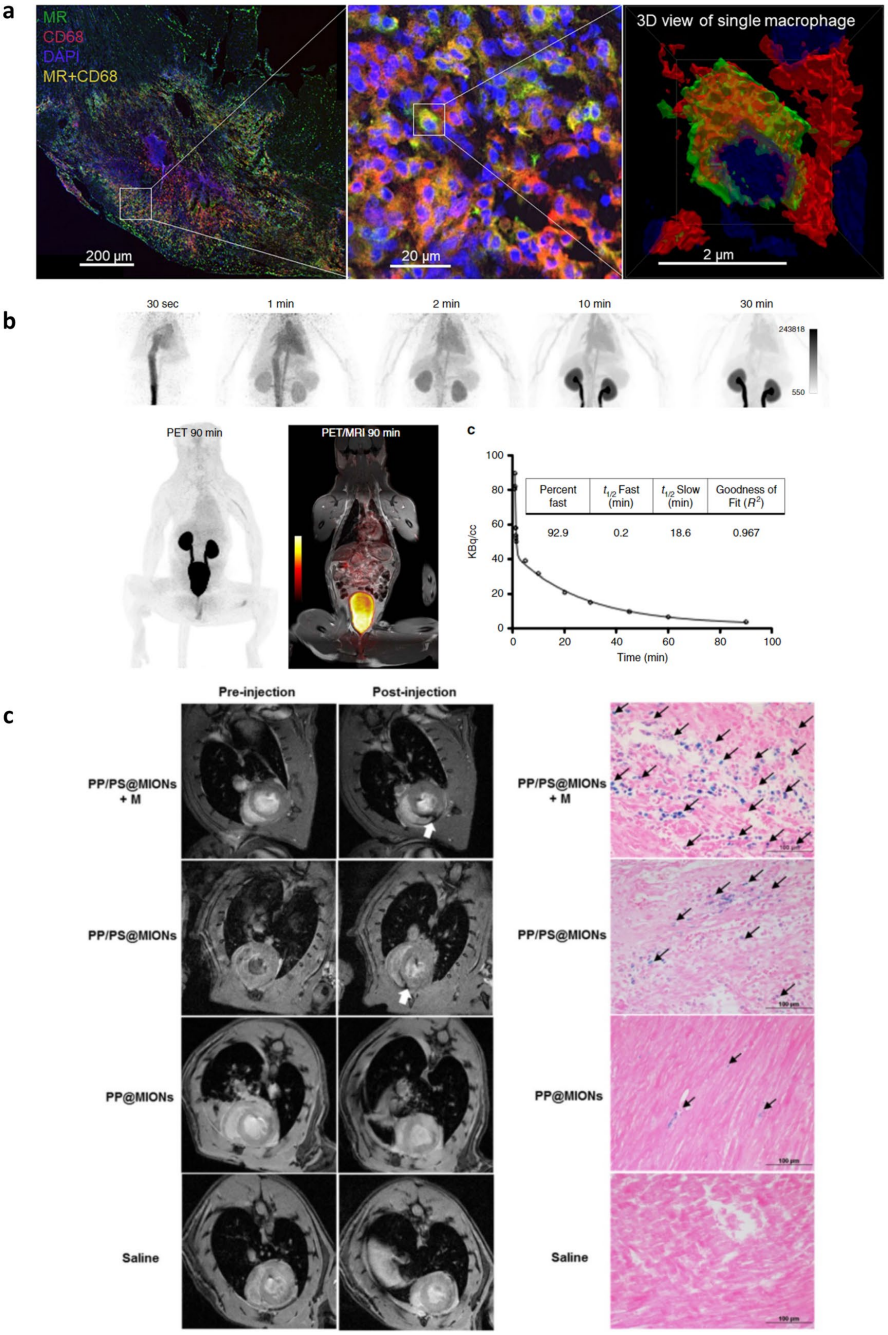
Nanomaterials and probes for imaging. **a** Confocal fluorescence images of ^68^Ga-NOTA-anti-MMR Nb, a nanotracer with specificity toward M2-like MF via mannose receptor (MR), uptaken in MF in the infarct zone 7 days post-MI. Figure reproduced from [138]. **b** PET/MRI of a non-human primate after administration of ^18^F-Macroflor over 90 min. The MF-targeted agent is rapidly cleared from circulation by renal excretion (half-life of 21.7 min) to enable subsequent whole-body imaging of MF abundance. Figure reproduced from [141]. **c** MRI (left) and corresponding histology (right) of infarcted rat hearts before and after injection of the theranostic iron oxide polymer nanocarriers (PP/PS@MIONs), showing MF-targeted accumulation that is further enhanced by application of an external magnetic field (+ M). Image reproduced from [143]

PET is another form of imaging used widely for cellular tracking. Inflammatory processes, including in the post-MI environment, can be imaged using ^18^F-FDG, which is preferentially accumulated in M1-like cells as a result of intracellular transport [133, 134]; concurrent suppression of cardiomyocyte glucose uptake is however necessary to reduce background in cardiac tissues [135]. To combat this, translocator protein (TSPO) may be used to assess inflammatory cell infiltration post-MI, including, for example, ^18^F-LW223 to map MF-driven inflammation post-MI [136]. TSPO-PET ligands may be uptaken preferentially by M1-like cells, but likewise accumulate in M2-like MF, neutrophils, Mo, T cells, and B cells to a lesser degree [133]. Using ^18^F-FDG in PET and MRI, one study characterized arterial inflammation in atherosclerosis. Results showed that uptake of the compound by MF was significantly higher in plaque-free arterial areas compared to the inside of plaques, suggesting that arterial inflammation does occur in early stages of atherosclerosis [137]. A ^68^Ga-NOTA-anti-MMR Nb tracer was designed to target mannose receptor on the surface of M2-like MF to demonstrate their abundance and localization in the infarct. Cell mapping with this nanotracer could therefore reveal a better understanding of the resolution of inflammation and predict cardiac remodeling outcomes post-MI (Fig. 3a) [138]. Macrin is a spherical polyglucose (i.e., dextran) nanoparticle, developed by Nahrendorf and colleagues. Using PET for quantitative assessment of cardiac MF, macrin was modified with ^64^Cu and used to treat mice, rabbits, and pigs. PET imaging indicated MF accumulation in the infarcted myocardium in all animals tested [139]. These demonstrate that ^64^Cu-macrin serves as an excellent nanotracer for MF, including for applications in cardiovascular medicine and quantitative assessment of tumor-associated MF (TAMs) [140]. The Nahrendorf lab also modified the macrin particle to create ^18^F-Macroflor and delivered it to non-human primates, mice, and rabbits, showing enrichment in cardiac and plaque MF (Fig. 3b) [141].

In some instances, these and other imaging probes may be used as a multifunctional tool for simultaneous therapy and diagnostics (i.e., theranostics) [142]. For example, Chen et al. developed a dual targeting theranostic system, PP/PS@MIONs, composed of magnetic iron oxide nanocubes for visualization via MRI that were enclosed in a zwitterionic copolymer, poly(lactide)-polycarboxybetaine (PLA-PCB, PP). Further surface modification by phosphatidylserine was used to modulate MF phenotypes. MRI showed PP/PS@MIONS accumulated in significantly greater amounts in infarcted tissue compared to other groups. Treatment also decreased the expression of pro-inflammatory markers, CD86, TNF-α, and IL-1β, and increased that of anti-inflammatory markers, CD206, TGF-β, and IL-10, in vitro (Fig. 3c) [143] and is an excellent example of theranostic systems that can simultaneously assess and treat underlying pathology. Quite separately, it is interesting to consider that simultaneous image-based evaluation and therapeutic intervention do not have to be administered as a single entity. The presented imaging modalities can additionally serve as companion imaging agents, administered concurrent with therapeutic delivery. Such techniques uniquely enable the real-time quantitative assessment of outcomes. While these techniques have been advanced in the area of cancer immunotherapies [144], their use in cardiovascular medicine has been less well explored.

## Therapeutic strategies

While appropriate physiological models and advanced imaging techniques allow for the study of inflammatory processes and their relationship to tissue-level processes, a wide variety of therapeutics (Table 2) allow for direct perturbation of the inflammatory response. Many of these tools are biotherapeutics, comprised of or derived from a biological source. These may include cells, cell-derived products (extracellular vesicles (EVs), antibodies, and cytokines), and even bioactive materials (collagen, decellularized ECM). In contrast, fully synthetic approaches such as small-molecule pharmaceuticals and synthetic biomaterials readily afford scalable production, tunable function, and greater control over batch-to-batch variability.

**Table 2:** Types of therapeutic cargo to aid in immunomodulation

**Therapeutic cargo**	**Considerations**	**Examples**	**References**
Cells	Cell sourcing, regulatory approval, and need for GMP processing with appropriate supply chains Potential for in situ recruitment or cell engineering may overcome barriers to production and therapy cost	Endothelial progenitor cells (EPCs) Stem cells MSCs ASCs BMSCs iPSC-derived cardiac progenitor cells CAR T	[145, 146] [147, 148] [149] [150] [151] [152]
Extracellular vesicles and exosomes	Require an abundant cell source for isolation Remain difficult to fully characterize Mechanisms of effect are yet to be better understood	BMSC-derived EPC-derived DC-derived MSC-derived	[153] [154] [155] [156]
DNA/RNA	Diverse structure and function Directly defined function Unstable, require vehicles for delivery Potentially immunostimulatory via TLR activation	miRNA siRNA mRNA siCCR2 siCRMP2	[157–160] [161, 162] [163] [164] [165]
Cytokines and chemokines	Direct biological signals with defined function Suitable for bioconjugation Required dosing may be unknown or difficult to achieve	IFN-γ IL-10 CSF-1 and IL-4 CCL5, CXCL12	[166] [167] [168] [169]
Small molecule drugs	Readily produced at industrial scale and amenable to synthetic modification Potential for oral bioavailability Frequent poor biodistribution May not require intracellular delivery, depending on drug target	Terpines Epinephrine Irbesartan Celastrol 1, 25-Dihydroxyvitamin D3 CCR2 antagonists Pitavastatin Atorvastatin	[170] [147] [171] [172] [173] [174] [175] [176]

Therapeutic strategies to address IHF progression can be either cardioprotective (often through immunosuppression) or immunoregenerative. The rationale for immunosuppressive therapies is to (i) attenuate leukocyte-mediated cardiomyocyte apoptosis that contributes to loss of contractility and border zone expansion, (ii) restrain protease activation to limit infract thinning and expansion, (iii) suppress fibrosis, and (iv) prevent secondary cardiac events (e.g., plaque rupture). Reparative strategies are a seemingly natural extension of cardioprotective therapeutics that seek to modulate the phenotype of cells in the infarct environment or selectively recruit progenitor cells to promote angiogenesis or other forms of myocardial regeneration. Here, we review cell-based and pharmaceutical approaches that have been implemented in pre-clinical and clinical studies, while EVs and naturally derived materials are later discussed.

### Cells

Cell therapies have been widely explored for HF treatment. Stem cell therapies have attracted particular attention, with early studies aimed at functional tissue replacement by cell differentiation. While claims of stem cell transdifferentiation into cardiomyocytes have been disputed [177, 178], they mediate the post-MI environment through a variety of signaling mechanisms to produce abundant growth factors, cytokines, microRNAs, and exosomes that constitute an immunomodulatory secretome [179–181]. It is through these paracrine signals that stem cell therapies modify immune cell recruitment and function [48, 182, 183], offering a means to both reduce detrimental inflammation while simultaneously promoting a switch towards tissue repair processes.

MSCs have become the prevailing cell type for HF treatment because they are pluripotent, genomically stable, and easily harvested from both mouse and human tissue [184–188]. MSCs modulate the inflammatory microenvironment of the myocardium via membrane receptors and a paracrine secretome that affect the migration, apoptosis, and phenotypic polarization of immune cells [48]. The specific interactions that exist between MSCs and immune cells are continuing to come to light. Although studies showing interactions between MSCs and neutrophils are sparse, Kang and colleagues reported a marked increase in neutrophil recruitment after MSC treatment. In the same study, MSC-conditioned media inhibited neutrophil apoptosis [189]. While neutrophils are crucial in post-MI repair, their overactivation can lead to reactive oxygen species (ROS) production and worsen injury [190, 191]; therefore, further studies are needed to understand these cellular interactions. MSCs have also shown direct effects on MF chemotaxis to the myocardium via signaling molecules such as CCL2, CCL7, and CCL12 [192, 193]. In another example, when MSCs were co-cultured with MF, the culture medium contained lesser amounts of pro-inflammatory markers, such as TNF-α, IL-1β, and IFN-γ, and greater amounts of anti-inflammatory cytokines, such as TGF-β and IL-10 [194, 195]. Even further, the MSC secretome contains prostaglandin E2 (PGE2), IL-1Rα, and TGF-β, all of which have been shown to guide M1-like to M2-like MF polarization [194, 196, 197].

MSCs can also mediate the cells of the adaptive immune response. MSCs express inhibitory signaling ligands that bind to complementary receptors on T cells and induce apoptosis, which halts T cell proliferative capacity, downregulates pro-inflammatory T cell populations, and abates the damaging state of the myocardium after MI [198, 199]. MSCs do not need to be in direct contact with T cells because they possess paracrine factors within their secretome, such as nitric oxide, TGF-β, and PGE-2, which prevent T cell proliferation and limit cellular impact on the infarcted heart [200–202]. In addition, culturing T cells with MSCs has resulted in the proliferation of FOXP3^+^ Tregs, which are crucial in propagating a reparatory state post-injury [203–205]. Although the known interactions between stem cells and B cells are limited, Che et al. found co-culture with MSCs to suppress B cell differentiation and proliferation [206]. ESCs have been shown to differentiate into M1-like and M2-like MF phenotypes and alter the inflammatory environment accordingly [207]. For example, Kudo and colleagues created an ESC-derived suppressor cell line containing a hybrid M1-like and M2-like MF phenotype that suppressed T cell responses [208]. When directly exposed to ESCs, CD3^+^ T cell populations within the myocardium increased, which induced Treg differentiation. However, due to the plasticity of Tregs, the resulting response tended to be heterogenous [209–211].

While stem cell-based therapies have largely focused on anti-inflammatory paracrine effects as mediators of LV remodeling, a critical alternative model of action has recently been proposed. Vagnozzi et al. proposed a comprehensive pro-inflammatory immunoregenerative hypothesis as the mechanism of therapeutic activity [212]. They compared the effects of locally injecting either mature stem cells or zymosan (a toll-like receptor 2 agonist) into healthy hearts, both of which produced transient accumulation of activated (CCR2^+^, CX3CR1^+^) MF at the site. When applied to the following ischemic injury, both treatments exhibited comparable improvements in cardiac function 2 and 8 weeks post-injection, relative to saline controls. These benefits were lost when mice were immunosuppressed or when MF were depleted, indicating that MF presence and activation were essential, including towards preferential alteration in ECM content and associated mechanical properties. Similar results were observed with injection of non-viable stem cells, ruling out potential paracrine signaling mechanisms. Taken together, these studies indicate that the transient accumulation of activated MF subtypes following immunostimulatory injection improves function of the injured heart by influencing cardiac fibroblasts.

Direct transplantation, including of naturally occurring or engineered immune cells, has also emerged as a cardioprotective treatment for MI. Such adoptive cell transfers using naturally occurring Tregs are an attractive approach [213], which has been leveraged by Sharir and colleagues to influence LV remodeling. The adoptive transfer of Tregs in mice reduced infarct size, attenuated LV remodeling, and improved heart function. Treg depletion using anti-CD25, however, had no effects on cardiac repair [214]. In vitro, Tregs are able to modulate Mo differentiation to a more anti-inflammatory subset. An in vivo myocarditis model was treated with Tregs, which showed cardioprotection against inflammatory damage and fibrosis through Mo modulation [215]. Meng et al. have further explored engineered cell therapies, wherein they induced MSC overexpression of IL-10 using CRISPR. Treatment showed increased IL-10 expression in the heart and decreased inflammatory cell infiltration, pro-inflammatory markers, and cardiac cell apoptosis, all of which improved cardiac recovery [216]. In recent groundbreaking work, the Epstein lab uniquely targeted cardiac fibrosis by the targeted elimination of myocardial fibroblasts, accomplished via adoptive transfer of CAR T cells active against fibroblast activation protein (FAP). Treatment significantly reduced cardiac fibrosis and partially rescued heart function in a mouse model of hypertensive cardiac injury [152].

The studies outlined here provide a basis for the beneficial effects of cell therapy on the post-infarct myocardium. The ability of stem cells to recruit immune cells to the injured area and facilitate modulation of their function is a promising methodology; though, the underpinning mechanisms are continuing to be better understood. Moreover, adoptive cell transfer is an exciting avenue for more targeted and intentional therapeutic outcomes. These cell therapies are at the cutting edge of cardioimmunology, particularly in the case of engineered effector cell types that uniquely enable discrete manipulation of the post-MI immune microenvironment through the targeted depletion of harmful cell types or the selective production of reparatory soluble signals. While such cell therapies are challenged by issues of cell sourcing, in vitro expansion, and need for the maintenance of supply chains in GMP processing, autologous cell therapies are among the fastest expanding markets for immunoncology [217]. Improvements to the in vivo lifetime of these cells and the ability to generate them directly in situ (vide infra, lipid-based nanoparticles) will continue to advance their road to the clinic.

### Biomolecules

Therapeutic biomolecules include a host of cell-derived products, ranging from proteins and antibodies to RNA and peptides. In many instances, these biological signaling molecules may be isolated components of a particular cell population’s secretome with well-defined immunological function. Of the biomolecules that are able to modulate the immune system after MI, proteins make up a significant contribution and include growth factors that can contribute to cardiac repair. Fibroblast growth factors (FGFs) facilitate a number of biological processes; Joki et al. injected FGF21 in a murine model post-MI and found that FGF21 exhibited anti-inflammatory properties (decreased TNF-α, IL-6), which attenuated remodeling and cardiomyocyte apoptosis while encouraging blood vessel formation [218]. Vascular endothelial growth factor (VEGF) has been extensively studied for cardiac repair, owing to its role in angiogenesis [219]. Rosano et al. delivered VEGF to the infarct in a rat MI model. The delivery of VEGF reduced collagen deposition, increased systolic function, and promoted microvascularization [220]. Bauza and colleagues investigated the effects of HMGB1, a non-histone chromatin binding protein and pro-inflammatory alarmin, on sheep with acute MI. Results showed that high-dose HMGB1 injection increased Ki67^+^ cardiomyocytes and overexpressed VEGF. This was accompanied with enhanced LV ejection fraction and wall thickening [221]. These outcomes are an interesting parallel to the earlier discussed treatments with zymosan, supporting the role of early pro-inflammatory interventions to promote reparatory processes in the injured heart.

Peptide therapeutics first emerged a century ago and have since become widespread in the field due to their low toxicity, high potency, and strong selectivity [222]. The cardioprotective potential of peptide therapeutics in MI has also become a field of interest. Qin et al. employed a glucocorticoid-regulated anti-inflammatory mediator, annexin-A1 (ANX-A1), and demonstrated its ability as a “triple shield” therapy, inhibiting neutrophil infiltration and preserving both cardiomyocyte viability and myocardial contractility [223]. Stromal cell-derived factor (SDF)-1α has previously been reported to improve vasculogenesis and cardiac function after MI. However, the bulky structure and short half-life are suboptimal for therapeutic use. Therefore, Hiesinger and colleagues developed a minimized peptide analog of SDF-1α and demonstrated improved ventricular function in a rat model of MI [224]. In an interesting example, a purified leech peptide was able to inhibit MF migration through mechanisms involving JNK and p38 MAPK pathways [225].

Under the umbrella of protein-based drugs, antibodies have also emerged as potential therapeutic strategies. Unfortunately, early clinical trials using antibodies to target glycoprotein receptors on the surfaces of immune cells did not show positive results. For example, Baran and colleagues investigated the efficacy of an anti-CD18 recombinant monoclonal antibody in a double-blind randomized trial. It was found that while the antibody was tolerated, cardiac end points, such as coronary blood flow or infarct size, were not improved [226]. In another example, antibody blockade of the CD11/CD18 integrin receptor was investigated. However, treatment resulted in no reduction of infarct size in patients with acute MI [227]. Pexelizumab, a humanized monoclonal antibody, binds to the C5 component of the complement cascade and has been implicated in apoptosis inhibition and leukocyte infiltration in experimental models [228, 229]. In a clinical trial with over 5000 patients, pexelizumab treatment showed no effects in improving acute MI [230]. Trials targeting interleukins released from activated immune cells also showed limited benefit. Abbate et al. conducted a pilot study using anakinra, a recombinant IL-1 receptor agonist. There were no significant differences between control and treatment groups when comparing the primary endpoint of LV end-systolic volume [231]. IL-6 has been shown to contribute to atherosclerotic plaque destabilization, which leads to MI [232]. Therefore, Kleveland and colleagues employed tocilizumab, a humanized anti-IL-6 receptor antibody, in a clinical trial for MI. Results showed little to no effects on attenuating the acute inflammatory response [233]. In the MRC-ILA Heart Study, an IL-1 receptor agonist was directly injected into patients with acute coronary syndrome. The study concluded that treatment showed some reduction in inflammatory markers; however, further studies would need to be conducted to confirm these findings [234]. The emphasis on cardiac end points only and the lack of elucidating biological mechanisms from these antibody treatments leave the reasoning for failed clinical trials open to interpretation, and the use of later discussed biomaterials to enhance biodistribution and cell targeting of biotherapeutics could be an attractive method to improve clinical outcomes.

### Small molecule drugs

Small molecule pharmaceuticals are frontline immunotherapeutics, with applications toward a myriad of chronic immune diseases [235]. Anti-inflammatory therapies have been widely employed in the context of IHF and are a mainstay of current medical management that have been the topic of recent and direct review [236, 237]. Notably, there remains ongoing concern that anti-inflammatory therapies alone may worsen outcomes by inhibiting inflammation-dependent repair mechanisms, including angiogenesis. As these topics have been recently and thoroughly reviewed, only a brief discussion of pro-regenerative immunotherapies is included here.

Resolvins are bioactive lipid mediators that have shown success in inflammatory resolution through interactions with surface receptors on leukocytes [238–241]. Resolvins primarily function to inhibit neutrophil and Mo migration, which can protect tissue against chronic inflammatory injury [242]. In an ApoE^−/−^ mouse model, resolvin D2 and maresin 1 treatment prevented atheroprogression by driving MF toward a reparatory phenotype [243]. Treatment with resolvin D2 shows increased myocyte numbers with decreasing levels of TNF-α, granulocyte MF colony-stimulating factor (GM-CSF), and neutrophil migration [244]. In another study, although resolvin E1 reduced expression levels of TNF-α and IFN-γ, MF infiltration to the atherosclerotic plaque did not decrease [245]. In a rat model of IR, resolvin E1 reduced leukocyte infiltration 4 h after reperfusion, concurrent with a reduction in infarct size [246]. The same group demonstrated decreased neutrophil infiltration and infarct size in another study centered around MI and depression in rats [247]. In a C57BL/6 J mouse model of coronary artery ligation, resolvin D1 limited neutrophil recruitment in the myocardium, decreased the expression of fibrotic genes, and reduced collagen deposition, all of which ameliorated fibrosis and stabilized the ECM [240].

Statins are promising agents because their anti-inflammatory properties are driven by a plethora of factors. For example, they can inhibit leukocyte migration through decreasing the expression of ICAM-1 and MCP-1 and modulate T cell activity through inhibition of Th1 chemokine receptors [248–250]. In addition, statins can reduce the release of C-reactive peptide, cytokines, chemokines, and adhesion molecules [248]. Shibasaki et al. investigated the effects of pitavastatin in ApoE^−/−^ mice, finding that arterial inflammation in atherosclerotic plaque was reduced [251]. Simvastatin administration to ApoE^−/−^ mice decreased the expression of HMGB1, VCAM-1, and MCP-1, in addition to reducing vascular inflammation and atherosclerotic lesions [252]. In one clinical trial, patients were treated with a high dose of atorvastatin and moderate dose of rosuvastatin. Regarding inflammatory activity, both treatments similarly reduced TNF-α and IL-6 [253]. Liu et al. conducted a clinical trial to test if atorvastatin is able to limit inflammation and improve cardiac function after MI. Drug administration demonstrated low levels of C-reactive protein and MMP9, with improvements in LV ejection fraction and heart function [254]. While statins possess anti-inflammatory activity, they have also been shown to promote a reparatory M2-like phenotype. In a rat model of MI, atorvastatin administration downregulated pro-inflammatory markers, such as IL-1β, TNF-α, and iNOS, and upregulated anti-inflammatory markers, such as Arg1, indicative of a shift from an M1-like MF phenotype to M2-like [255].

Other small molecule drugs have also shown efficacious results in modulating the inflammatory response post-MI. Cyclophosphamide administration in a rat IR model, for example, resulted in lower rates of leukocyte infiltration and reduced the propensity of ventricular dysfunction [256]. In the COLCOT clinical trial, colchicine, an anti-inflammatory drug targeting MF migration, lowered the risk of adverse cardiac events in post-MI patients [257]. Pyruvate kinase isozyme type M2 (PKM2) is an enzyme in the glycolytic pathway that regulates inflammation in LPS-activated MF [258]. Iminostilbene, a modulator of PKM2, was shown to suppress levels of pro-inflammatory markers, such as IL-1β and IL-6, reduce infiltration of CD86 MF, reduce the phosphorylation of the STAT3 inflammatory pathway, and alleviate cardiomyocyte apoptosis in vitro and in vivo [259].

These and other immunomodulatory drugs are often identified by drug screening processes. For example, Hu et al. performed high-throughput drug screens of approximately 4000 compounds across a variety of drug classes to identify targets for MF modulation [260]. Currently, large drug screenings like this primarily focus on M1-polarizing agents, with limited success in identifying or following up on M2-promoting compounds. More recently, combinatorial drug screens combining immunostimulatory and immunosuppressive drugs or multi-phase screens have been used to identify pharmacological promoters of tolerogenic DCs and M2-like MF, respectively [261, 262]. Continued developments in drug screening methods, such as automated drug screening and methods to directly assay for M2-like promotion, will continue to move this field forward. As for antibody-based strategies, clinical trials have shown limited benefits to date and may gain better insights through the addition of secondary outcomes that include assessment of LV remodeling and inflammatory mediators.

## Nanomaterials

**Fig. 4:**
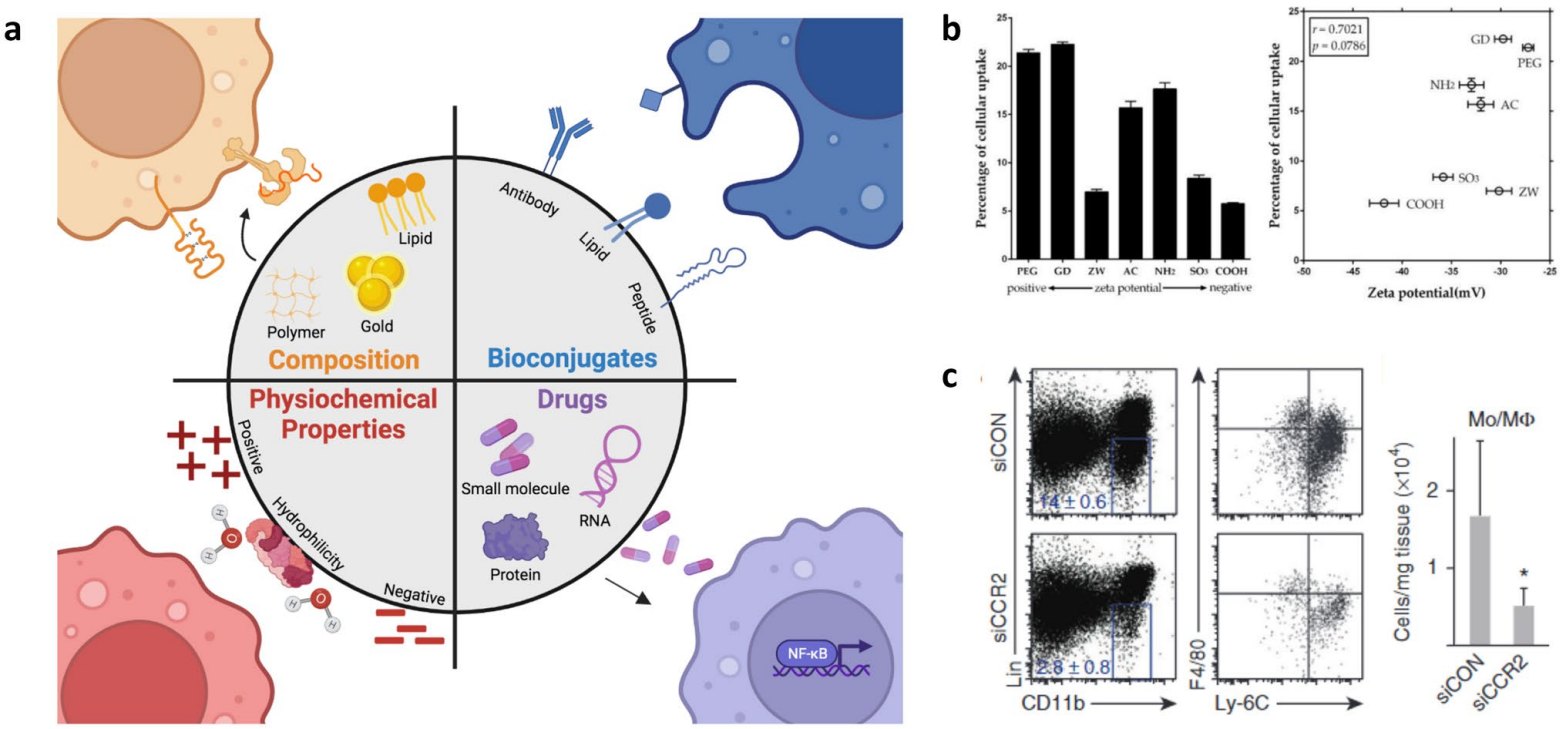
Diversity of immunotherapeutic nanomaterials. **a** Composition, properties, and therapeutic cargo dictate how nanoparticles interact with immune cells. These aspects enable cell-targeted delivery, receptor-mediated control of cell programs, and influence over downstream effector or suppressor signaling programs. **b** Lipid nanoparticles, synthesized with varying surface charges, were incubated with human MF in vitro; surface charge positively correlated with cell uptake. Figure reproduced from [283]. **c** siRNA loaded particle treatment (siCCR2) silences CCR2 to reduce inflammatory Mo infiltration and MF populations compared to the control (siCON) following IR injury. Figure reproduced from [164]

While therapeutics alone are clearly efficacious tools for reorientation of the post ischemic inflammatory milieu, their use can be hindered by factors such as suboptimal pharmacokinetics (i.e., rapid blood clearance, non-specific cell and tissue biodistribution) and resulting off-target effects such as systemic toxicity and increased risk of infection [263]. Functional biomaterials have been widely used to address these challenges in cardiac repair [264, 265], which may be composed of either natural or synthetic components (Table 3). Here, we outline biomaterial-based strategies, including systemically or locally administered therapeutic vehicles that have demonstrated utility in modulating the immune response to mitigate impacts toward IHF. However, it is worth noting that biomaterials themselves can have a profound effect on immune cell behaviors (Fig. 4), including via material composition or surface properties [266]. Here, we briefly review these nanoparticle properties in the context of IHF; the following sections focus on the use of these systems as drug carriers, used primarily for systemic administration to enable cell-targeted delivery.

**Table 3:** Material compositions used in both nanotherapeutic and bulk materials

**Material composition**	**Considerations**	**Examples**	**References**
Natural	Often widely regarded as biocompatible and biodegradable May include native functions, such as sites for cell adhesion or immune modulation by defined receptors Often well suited for modification by bioconjugate reactions For general discussion, see [309, 310]	Cardiac ECM Splenic ECM Hyaluronic acid Collagen Silk protein Alginate Chitosan Silica nanoparticles	[149, 311–314] [315] [151, 156, 316] [65, 317] [274] [153, 155, 168, 318] [150] [157]
Synthetic	Allow for user-defined physical and chemical tunability Require direct synthesis and purification For general discussion, see [319, 320]	PEG-based micelles, hydrogels PLGA nanoparticles Zwitterionic co-polymers Bioactive co-polymers Lipid nanoparticles	[148, 172–174] [171, 175] [143] [169, 321] [161–165]

### Nanomaterial properties

In the case of polymeric materials in particular, specific receptor-mediated interactions with immune cells can occur that are critical to immune engineering and biomaterial design [267]. Hyaluronic acid (HA), a glycosaminoglycan prevalent in the ECM, interacts directly with CD44, CD168 (receptor for HA-mediated motility, RHAMM), and toll-like receptors (TLRs). These interactions are critical toward neutrophil recruitment and MF polarization and are highly dependent on polymer molecular weight [268, 269]. Molecular weight of HA has been shown to have varying MF polarization potential and cardioregenerative effects [270, 271]. For example, 50 kDa, 130 kDa, and 170 kDa HA hydrogels were used to treat experimental MI, with 50 kDa HA exhibiting the greatest myocardial regeneration and functional recovery [272]. Wang et al. investigated short-chain HA fragments (6–10 disaccharides) that decreased the inflammatory response caused by neutrophils and facilitated MF polarization to the M2-like phenotype in a mouse MI model [273]. In an interesting example of proteinaceous hydrogels, Song and colleagues formed an injectable hydrogel from sericin, a silk-derived protein. In a mouse model of MI, the hydrogel downregulated the expression of inflammatory markers, such as TNF-α and CCL2, by suppressing TLR4/NF-κB pathways and ultimately decreased the number of MF in the infarct region by 45.8% [274].

Surface modification of materials is also a useful technique for targeting immune cells and modulating their response. Seminal work in this area of cardiac immune engineering was performed by the lab of Smadar Cohen and used phosphatidylserine-presenting liposomes to mimic apoptotic cell endocytosis, in turn polarizing MF towards an M2-like state [275]. Specific pathways can be ingeniously targeted by such methods, and deoxyribozyme (a DNA enzyme able to silence TNF-α) was conjugated to gold nanoparticles because DNA structures are easily internalized by nucleated cells [276]. After injection in a mouse model of acute MI, TNF-α levels were knocked down by 50% which better maintained cardiac function [277]. Richart and colleagues created nanoparticles made of apolipoprotein AI reconstituted with phosphatidylcholine (n-apo AI), which resembled high-density lipoprotein particles. Following MI, n-apo AI administration decreased the expression levels of chemokines that facilitate leukocyte recruitment by 60–80%, thus reducing the numbers of neutrophils and Mo in the myocardium and attenuating inflammation [278]. In another example, researchers developed α-gal epitope nanoparticles to incite recruitment of reparatory MF via activation of complement cascade and corresponding chemotactic cues. This was further confirmed by the repopulation of cardiomyocytes and restoration of normal cardiac structure and contractile function in the mice, suggesting a truly regenerative rather than cardioprotective treatment [279].

Surface chemistry and topography of biomaterials has likewise demonstrated distinct influence on contacting cells [280]. For example, neutrophils secrete greater levels of pro-inflammatory cytokines when in contact with hydrophobic surfaces [281], and surface roughness induces greater neutrophil death and ROS production [282]. MF has been likewise studied in this context, in part due to their prominent role in the foreign body response (FBR). Surface charge can have a substantial role in interactions with MF. For example, the surface modification of polystyrene nanoparticles has been used to demonstrate MF uptake in vitro is directly correlated with the surface zeta potential (Fig. 4b) [283]. Hamlet and colleagues demonstrated that hydrophilic surfaces decreased pro-inflammatory cytokine expression in human and mouse MF [284, 285], and similar outcomes have been noted for DCs [286]. In both in vitro and in vivo studies, increasing stiffness of the substrate is associated with a higher prevalence of the M1-like MF phenotype [287, 288], whereas softer surfaces are associated with a lesser FBR and fibrous encapsulation [289]. MF complexity has shown mixed results regarding surface roughness [290, 291], and recent results suggest that governance by Tregs may be responsible for such behavior in vivo [292]. Nanoparticle shape can also be readily tuned [293], providing unique opportunities to adjust how specific cell types interact with these materials. For example, elongated nanoparticles are preferentially uptaken by neutrophils as compared to other innate immune cells, providing the ability to discretely target these drug carriers [294]. These findings indicate the necessity of designing immunomodulatory devices and delivery systems with material composition and structure in mind. By doing so, it is possible to harness these aspects of cell-material interaction to not only aid in cell-targeted therapeutic delivery but also to improve treatment outcomes by the rationale design of drug carriers that complement or synergize with the action of encapsulated therapeutics.

### Polymeric nanoparticles

Nanoparticles are valuable drug delivery vehicles [295], most frequently used for systemic administration. They are well suited to the encapsulation of various small molecule drugs, RNA, and other biomolecules with dependence upon nanoparticle structure and material selection. Nanoformulation of cargo is particularly useful to improve bioavailability by enhancing drug solubility, preventing rapid renal clearance, and shielding sensitive cargo (e.g., proteins, nucleic acids) from enzymatic degradation [296, 297]. Furthermore, these systems have the potential to target delivery to specific cells or tissues via surface ligand modification, minimizing off-target exposure [298, 299]. Here, we will review the use of polymeric nanoparticles in delivering a variety of cargo, including small molecule drugs, mRNA, and others.

As discussed above, a plethora of immunoactive small molecule pharmaceuticals exist at varying stages of development and exploration towards cardiovascular engineering. As many of these are hydrophobic small molecules, they are amenable to encapsulation in polymeric nanoparticles, such as by nanoprecipitation and emulsion methods. Irbesartan is an angiotension II type I receptor blocker with a PPARγ agonist effect. In a murine IR model, PLGA nanoparticles loaded with irbesartan demonstrated inhibited recruitment of inflammatory Mo to the heart, reduced infarct size via PPARγ-dependent mechanisms, and improved LV remodeling after 3 weeks [171]. As mentioned, statins have also demonstrated cardioprotective effects. Pitavastatin-loaded nanoparticles were intravenously injected into C57BL/6 mice with permanent coronary ligation. Nanoparticles were uptaken by CD11b^+^ Mo/MF and reduced their prevalence in the infarcted heart and spleen, which ultimately attenuated LV remodeling [175]. In another example involving statins, atorvastatin loaded supramolecular copolymers demonstrated cellular drug uptake in MF and an increased ratio of M2-like to M1-like presence by 6.3-fold in an in vitro cholesterol model [176, 300].

### Lipid-based nanoparticles

Micelles are nanosized spherical vesicles composed of a lipid monolayer. During self-assembly, micelles form a hydrophobic core, which allows for incorporation of hydrophobic drugs [301]. These systems have been widely used to modulate the immune microenvironment post-MI. Allen et al. loaded celastrol, a small molecule immunotherapeutic, into poly(ethylene glycol)-b-poly(propylene sulfide) (PEG-b-PPS) micelles. Celastrol-loaded micelles reduced secretion of TNF-α in RAW264.7 cells in vitro, and their delivery decreased neutrophil and Mo recruitment to atherosclerotic plaque in *LDLR*^−/−^ mice [172]. Wang and colleagues developed PEG-based micelles loaded with a small molecule CCR2 antagonist and surface decorated with an anti-CCR2 antibody for cell targeting. Treatment in a murine MI model significantly decreased the number of Ly6C^high^ inflammatory cells compared to the control group, while also reducing infarct size [174, 302].

Liposomes are among the first nanoformulations to be clinically used because of their amphiphilic composition that promotes encapsulation of hydrophobic drugs in the lipid bilayer and hydrophilic drugs in the aqueous cavity [175]. In a rat model of acute MI, intravenous injections of phosphatidylserine-presenting liposomes upregulated the expression of anti-inflammatory cytokines, such as TGF-β and IL-10, increased the number of anti-inflammatory CD206^+^ MF, and decreased the levels of pro-inflammatory markers, such as TNF-α and CD86 [275]. In a similar study that employed the same type of liposomes, researchers were able to upregulate the expression of anti-inflammatory genes, while downregulating the expression of pro-inflammatory genes for infarct repair in vivo [303].

The use of RNA as a therapeutic cannot be understated given the success of the mRNA-based vaccines for the recent COVID-19 pandemic. Because many diseases, like cancer and immune disorders, have discrete genetic targets, delivery of RNA is a feasible strategy for treatment, and various RNA therapeutics have been explored in regenerative medicine [304, 305]. However, the delivery of RNA alone is susceptible to rapid degradation and off-target effects [306]. Encapsulation is therefore useful to safely carry RNA to sites of interest [307]. In targeted applications for IHF prevention, siRNA has been widely explored both in ischemic injury and atherosclerosis. For example, collapsin response mediator protein-2 (CRMP2) was shown to be involved in MF polarization; therefore, Zhou and colleagues loaded siCRMP2 into lipid nanoparticles (LNPs), finding MF polarization from M1-like to M2-like, decreased inflammatory and fibrosis markers, and attenuation of LV remodeling in both WT and ApoE^−/−^ mice [165]. Courties and colleagues likewise identified high levels of interferon regulatory factor 5 (IFR5) expressed by inflammatory MF after injury; siIRF5 delivery reduced the expression levels of M1-like MF markers, supported inflammation resolution, and promoted infarct healing [161]. In another example, LNPs carrying siCCR2 were shown to accumulate in splenic phagocytic cells and localized to Mo when administered to mice. This treatment significantly decreased the level of inflammatory Mo and MF in atherosclerotic plaque in the ApoE^−/−^ model and reduced infarct size following coronary artery occlusion (Fig. 4c) [164]. In yet another study by the Nahrendorf group, siRNA targeting five different cell adhesion molecules were loaded into a single endothelial cell targeted polymeric nanoparticle. Treatment in the ApoE^−/−^ and coronary ligation models attenuated leukocyte recruitment to these sites and improved outcomes [162, 308].

While many RNA delivery strategies have focused on silencing specific targets of interest, the same means can be used for cell and gene therapy to promote immunoregulatory behavior. In an exciting example led by the Epstein lab, earlier discussed methods of CAR T therapy have been recently adapted to in situ cell therapies, eliminating the need for initial cell isolation and adoptive transfer [163]. In this work, mRNA necessary for CAR T reprogramming was encapsulated in CD5-targeted LNPs, enabling the transient in vivo generation of FAP CAR T cells that reduced fibrosis and restored cardiac function after injury. Biomaterial-based strategies such as these are invaluable advances in the field—they hold promise to revolutionize the face of cell and gene therapies by eliminating the time and labor-intensive supply chain required for cell manufacturing. By performing these cell manipulations directly within the body, these therapeutic strategies are reduced to a cost-effective off-the-shelf approach that is more accessible to broad use.

### Biologically derived nanoparticles

Within the body, cells release EVs through endosomal pathways and budding from the plasma membrane. These naturally arising nanoparticles contain RNA, proteins, and other soluble or membrane-bound factors that are fundamental to understanding, as well as manipulating intercellular communication [322]. Immune cells continually exchange EVs as part of the dynamic network of communication among the innate and adaptive immune compartments. For example, activated DCs express co-stimulatory CD80 and CD86; their secreted EVs can therefore activate T cells [323]. Furthermore, miRNA-loaded EVs transferred from Tregs to Th1 cells have been shown to reduce the Th1-driven inflammatory response [324], and MSC-derived EVs suppress inflammatory MF activation through modulated NF-κB pathway signaling [325]. Cardiac-derived cells (CDCs) similarly mediate the polarization from an M1-like to M2-like MF phenotype as well [161, 326, 327]. As such, the injection of CDC-derived exosomes demonstrated an increase in anti-inflammatory gene expression, accompanied by a decrease in pro-inflammatory expression [328].

The mechanisms of these effects in cardiac tissues have been both explored and manipulated for therapeutic benefit. The cardioprotective benefits of MSC-derived exosomes, for example, have been associated with miR-182 content, a potential mediator of MF polarization and TLR4 expression [158]. MSC-derived EVs have also been purposefully loaded with exogenous miR-101a to target TGF-β and Wnt signaling and attenuate fibrosis [159]. M2-like MF, which were programmed to secrete miR-148a exosomes, were shown to reduce infarct size and improve cardiac function post-MI in vivo [160]. Wu and colleagues engineered M2-like MF exosomes with hexyl 5-aminolevulinate hydrochloride (HAL), an FDA-approved imaging agent that has been shown to initiate the production of anti-inflammatory compounds. The system exhibited anti-inflammatory capabilities and reduced progression of atherosclerosis [329]. While these examples display the complex role of EVs as well as their potential as tunable immunotherapeutics for CVD, a full understanding of their mechanistic origin and functionality remains lacking and will no doubt contribute to further advances in this exciting area.

## Bulk materials and devices

**Fig. 5:**
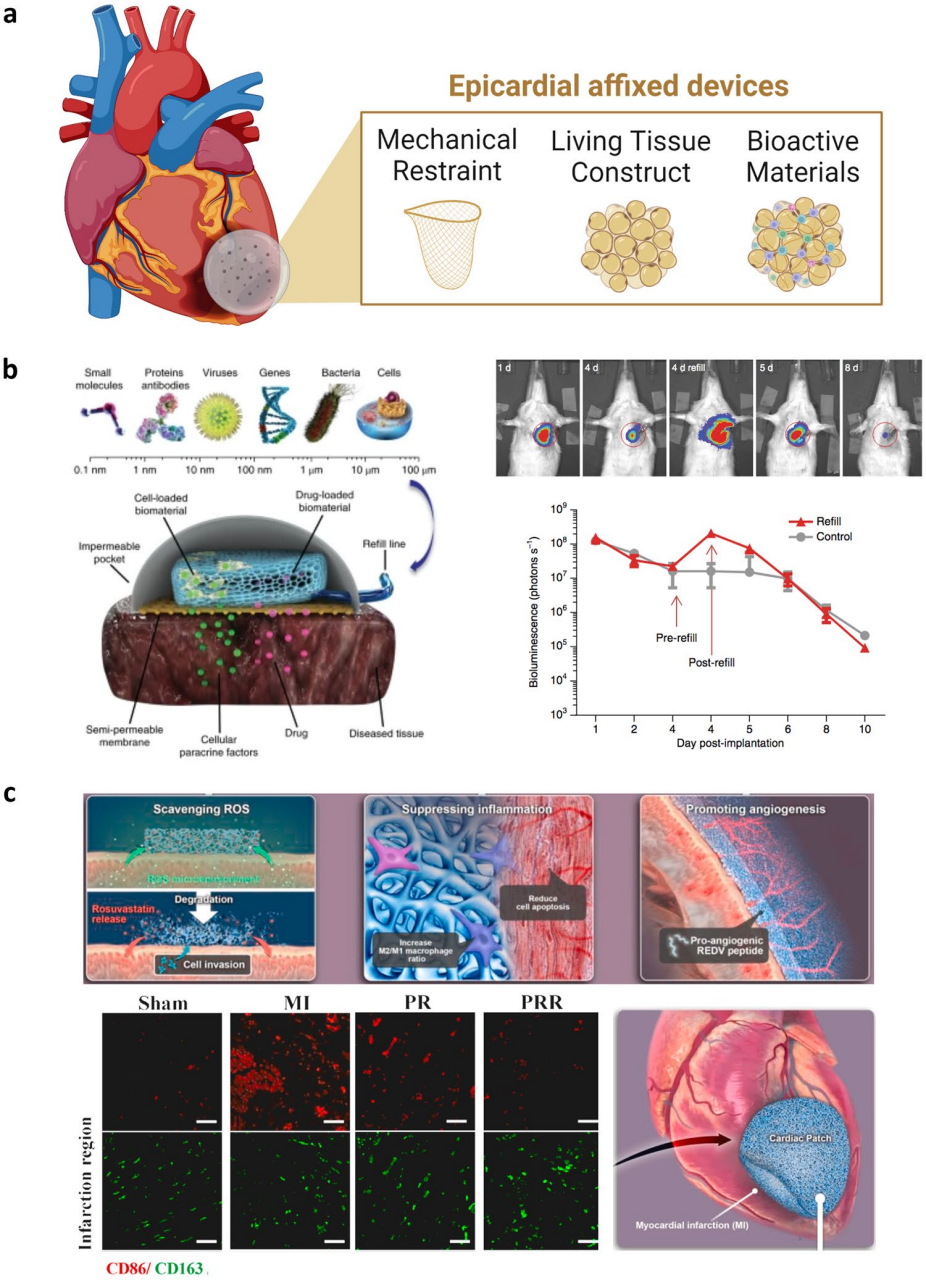
Epicardial affixed devices such as patches and wraps allow for mechanical stabilization of the infarct and can simultaneously deliver therapeutics or incorporate bioactive materials. **a** The evolution of epicardial affixed devices initiated with mechanical restraints to prevent LV dilation and has moved to incorporate living tissue constructs and bioactive materials for immunomodulation. **b** Schematic of the Therapi system, which incorporates a semipermeable membrane in contact with the heart surface and a delivery reservoir, replenishable via an externally accessible refill port. Luciferase-expressing MSCs were loaded before implantation (control) and optionally re-filled (day 4). Figure reproduced from [147]. **c** Synthesis of PTFU (an ROS scavenger) combined with PTK and PPF is clicked with pro-angiogenic REDV peptides to create a multifunctional macroporous cardiac patch. The cardiac patch is further loaded with rosuvastatin and surgically implanted onto the LV ischemic areas of rat hearts in an acute MI model. In vivo, the patch acts as a ROS scavenger and regulates MF phenotype. Figure reproduced from [321]

**Fig. 6:**
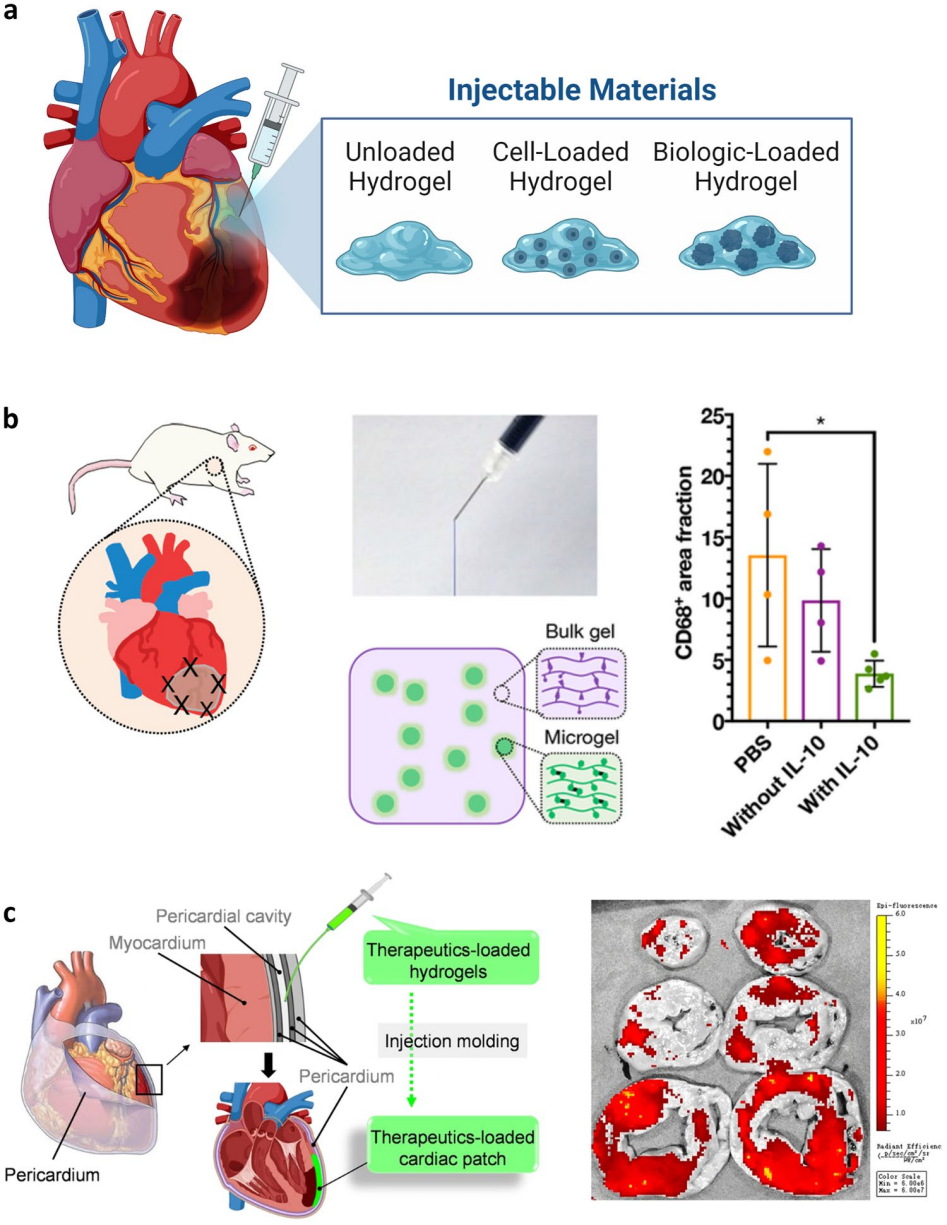
Injectable materials, such as hydrogels, potentiate minimally invasive and local delivery of therapeutic cargo to the heart via intramyocardial or pericardial injection. **a** Hydrogels loaded with cells and biologics can be delivered to aid in immunomodulation, while the hydrogels themselves provide needed mechanical restraint of the infarct. **b** Shear-thinning Ad-HA and CD-HA hydrogels including IL-10-loaded NorHA microgels were injected into the border zone of the infarct in a rat MI model. Local delivery of IL-10 decreased CD68^+^ MF after 1 week. Figure reproduced from [167]. **c** The pericardial space acts as a natural mold for hydrogels to form a cardiac patch in situ and release loaded therapeutics. Pericardial injection of methacrylated HA hydrogels with MSC-derived exosomes in pigs increases exosome retention in the heart and offers a local and minimally invasive delivery approach. Figure reproduced from [151]

While nanotherapeutics have emerged as critical drug delivery vehicles for cell-targeted delivery, they rarely allow tissue-specific tropism and therefore do not address issues of off-target drug effects such as systemic immunosuppression. Towards this goal, bulk biomaterials and devices have advanced in a parallel manner and offer additional means of mitigating LV remodeling—such as infarct restraint to prevent infarct or LV dilation. For the development of biomaterial strategies to treat MI, multiple design factors must be considered. These include the intended therapeutic payload, material composition, and overall structure—each of which can influence the immune response either inadvertently or for intended effects. As bulk materials are locally applied interventions, the device structure and route of introduction must also be considered. Frequently employed methodologies are myocardial wraps and patches surgically applied to the epicardial surface (Fig. 5) and injectable biomaterials that may be applied by coronary perfusion, intramyocardial injection, pericardial injection, or other means (Fig. 6). Wherever possible, minimally invasive routes of introduction are preferable to open thoracotomy. These considerations are in large part due to procedural complexity, cost, and associated risks of morbidity and mortality. Moreover, it is increasingly realized that surgical stress profoundly influences the systemic immune environment, with implications in both cancer progression and postoperative cardiovascular events [330–332].

### Externally affixed devices

Externally affixed myocardial wraps and patches are a form of mechanical stabilization for post-MI treatment, originally intended as a prophylactic strategy to directly prevent infarct expansion and LV dilation. These early biomaterial-based interventions are referred to as ventricular restraint devices (VRDs), and often use a wrap or patch to mechanically restrain the heart [333–335]. One type of VRD arose in the form of a cardiac wrap to enable diastolic reinforcement, called the CorCap Cardiac Support Device (CSD, Acorn Cardiovascular). The CSD is a polyester mesh that fits around the ventricles to reduce wall stress and prevent LV dilation. The device was shown to reduce stress response proteins, attenuate cardiomyocyte hypertrophy, and normalize MHC isoforms contributing to improved myocardial kinetics in large animal models and long-term benefit in clinical trials at 5-year follow-up [336–338]. Progress towards similar devices have included the HeartNet (Paracor Medical, Inc.) [339], which is placed around the heart with an introducer sheath via minithocotomy as well as the quantitative ventricular restraint device (QVR; Polyzen Inc.) that incorporates an inflatable balloon structure and access line for adjusting the heart volume and pressure [340]. Advances in VRDs and related direct cardiac compression devices, as well as their relative advantages and disadvantages, have been the topic of focused review by Naveed et al. [335, 341].

Since their conception as VRDs, externally affixed devices have further evolved to incorporate immunotherapeutic payloads, bioactive materials, and living tissue constructs. In particular, the active hydraulic ventricular attaching support system (ASD) device was designed as a multi-purpose device. The ASD is composed of a mesh cover that incorporates silicone tubes, accessible via an external port for injection. The tubes can be used to locally deliver therapeutic drugs [170], as well as to apply altered pressure to tune LV restraint similar to the QVR [342]. Likewise, another device of note is the Therapi system that was designed as an epicardial reservoir, amenable to minimally invasive implantation and refillable loading for the local and sustained presentation of therapeutic cargo [147]. The system is placed in the epicardium, where a subcutaneous catheter port allows for local delivery of therapeutics and a minimally invasive refillable component (Fig. 5b). The delivery of small molecules (epinephrine), macromolecules (dextran and albumin), and cells (MSCs) demonstrated a range of potential cargo. Repeated cell dosing post-MI better maintained heart function, suggestive of the intended therapeutic benefit.

While ventricular restraints (wraps) are more efficacious than infarct stiffening (patches applied to the infarct area alone) [343], cardiac patches are likewise a means of myocardial restraint that can be readily tuned in composition and payload. Patches can be formed from natural (e.g., polysaccharides, decellularized ECM, proteins) or fully synthetic (e.g., PLGA, PLLA, PEG) starting materials, each of which have their own benefits [344–346]. Natural biomaterials often lend themselves towards mimicking the mechanical properties, resorption behavior, and cell interactions of native tissues, whereas synthetic polymers or modifications readily enable tuning of these and other material properties [267, 320, 347]. One promising method for cardiac patch creation incorporates the use of the decellularized ECM because it contains proteins and proteoglycans that can allow for cell attachment and proliferation to facilitate cardiac repair [348, 349]. Sarig et al. investigated the use of a decellularized porcine cardiac ECM patch (pcECM-P), applied to Wistar rats in either the acute or chronic inflammatory phase. The patch induced constructive remodeling, attributed in part to a stark increase in the M2/M1-like MF ratio that was associated with enhanced vascularization and cardiomyocyte differentiation markers [311]. Further work by Ge Zhang and colleagues has used decellularized porcine myocardium slices (dPMS), either as an acellular patch or after seeding with adipose-derived stem cells (ASCs) [149, 312]. At 4 weeks post-MI in rats, acellular dPMS treatment was associated with a more robust MF infiltrate and markedly higher M2/M1-like ratio that was associated with increased vascular density and better preserved fractional shortening. Both rat and pig ASCs were readily able to infiltrate the matrix by seeding in vitro, improving their local retention as compared to direct injection. Such approaches are a promising strategy to encompass infarct restraint, modulation of host immunoregenerative response, and cell therapies within a single approach.

By the inclusion of other cell types, living tissue constructs can also be formed as cardiac muscle patches (CMPs) to treat HF. CMPs can not only assist in ventricular contraction, but also potentially contribute to molecular and electrical signaling. Cardiomyocytes, cardiac vascular cells, and fibroblasts have most commonly been used [350]. However, one study composed a bi-layer cardiac patch from hiPSC-derived cardiomyocytes and a sheet of blood outgrowth endothelial cells (BOECs) and pericytes (PCs). Grafting in a nude rat infarct model attenuated infarct fibrosis and thinning, in part due to microvascular connection between the graft and host tissue [351]. Similarly, Weinberger and Querdel et al. created hiPSC-derived cardiomyocyte patches from 3 separate hiPSC lines and studied outcomes in guinea pigs and pigs. In guinea pigs, the patch formed heart muscle, improved electrical function, and increased LV function. When used in pigs, there was successful transplantation and evidence of cardiomyocyte proliferation, suggesting a promising method for post-MI healing [352, 353]. Given the newly established role of cardiac MF in electrical conduction in the heart, it is possible that inclusion of such cell types may further enhance electrical connectivity of CMPs and the native tissue, which has remained an ongoing challenge of the field. Additionally, these fields highlight a need for continuous pharmacological immunosuppression to be used for certain cell sources.

Through further tuning of material composition and cell or drug cargo, cardiac patches can be further developed to directly modulate the post-MI environment [346]. Hosoyama et al. have reported the development of a bilaminar cardiac patch, composed of an elastic hydrodynamic support coupled with aligned electrospun collagen that contained silver (AgNP) or gold (AuNP) for electroconductivity. Interestingly, only AuNP-containing patches preferentially skewed the M2/M1-like phenotypic ratio towards the creation of a reparatory environment [317]. In another excellent example of synthetic adaptations to develop multifunctional and immunomodulatory patches, elastomeric cardiac patches were prepared to restrict LV remodeling. The patch was composed of polyurethane and unsaturated poly(thioketal) (PTK, as an ROS scavenger), further modified by a pro-angiogenic peptide (REDV) and incorporated rosuvastatin (Fig. 5c). In addition to provision of mechanical stabilization, the multiple precise modifications were intended to suppress early inflammation, foster angiogenesis, and prevent fibrosis throughout the stages of LV remodeling. In a rat model of MI, the multifunctional support effectively increased M2-like MF at the infarct site and downregulated genes associated with IFN-γ production and TGF-β signaling [321]. While MF modulation remains a mainstay of cardiac immunotherapies, other studies have leveraged an understanding of T cell response. Across CVD and other diseases, the recruitment and differentiation of Tregs is a valuable immunomodulatory mechanism [354, 355]. Ramjee et al. demonstrated that Treg recruitment post-MI is dependent on Hippo signaling, and inflammatory cardiomyopathy and death are therefore exaggerated by disruption of core pathway effectors (YAP/TAZ). Delivery of IFN-γ by formation of a hydrogel patch photopolymerized directly onto the epicardial surface rescued Treg infiltration and reversed deleterious inflammation [166]. This approach represents a divergence from conventional approaches. While many methods focus on suppression of the innate immune response including downregulation of IFN-γ production, these inflammatory mediators are likewise a valuable tool for regulating the downstream adaptive immune compartment (B and T cells). These studies demonstrate the multitude of methods, spanning from electrical signaling to emerging therapeutic targets, that can be used to modulate post-MI inflammation.

### Injectable materials

Another common form of cardiac therapies is the use of injectable hydrogels, which are an alternative means of mechanical support that enable local and minimally invasive delivery to the heart [334, 356]. Alginate has been widely investigated in pre-clinical large animal models, where introduction can be performed by intracoronary or intramyocardial injection to attenuate LV remodeling [357–360]. This line of work has progressed to clinical trials (AUGMENT-HF; NCT01311791, NCT03082508), where Algisyl-LVR improved clinical outcomes in patients with advanced HF [318]. Additional investigations through the use of synthetically tunable materials have continued to demonstrate through a combination of experimental and computational approaches that supraphysiological material stiffness, hydrogel injection volume, and even injection location can further improve these outcomes [361–366].

As for myocardial wraps and patches, mechanical restraint is a primary mechanism of therapeutic action; however, bioactive roles are also critical. Again, decellularized ECM has become often employed due to its bioactive role [367]. The Christman lab has pioneered this work by developing techniques to process decellularized porcine myocardial ECM into an injectable form, amenable to catheter-based injection [313, 368]. While these and related studies indicate only a moderate effect on MF infiltration and polarization state, an overall effect on inflammatory pathway activation has been noted [314]. A clinical trial arising from this work (Ventrix, Inc.; NCT02305602) indicated moderate increases in clinical outcomes, and no incidence of adverse events was definitively linked to VentriGel injection [314]. Other source tissues have also been explored. Inspired by its immunological function, Liu et al. used hydrogels derived from spleen ECM [315]. At physiological temperatures, the materials self-assembled into a hydrogel that drove an anti-inflammatory MF phenotype that was recapitulated in vivo*.* Improvements in both lymphangiogenesis and heart function were noted following MI treatment.

Self-assembling hydrogels are also useful as vehicles for therapeutic delivery. Rodell et al. have developed injectable hydrogels based on guest–host associations that can be pre-formed, injected via shear-thinning processes, and rapidly reassembled in the tissue [316]. Secondary covalent crosslinking interactions were used to achieve supraphysiological moduli for mechanical restraint [363, 369]. Alternatively, the shear-thinning materials alone have been used for the delivery of bone marrow cell chemotaxis enhancers and endothelial progenitor cells (EPCs) [145, 146]. Even though these hydrogels can be delivered alone, they are also convenient for cytokine and chemokine delivery. A follow-up study subsequently delivered anti-inflammatory cytokine IL-10 from a supramolecular HA hydrogel/microgel composite in a rat MI model. Delivery of exogenous IL-10 significantly decreased MF infiltration at 1-week post-treatment and improved vascularization and heart function at study endpoint (Fig. 6b) [167]. Alginate hydrogels were similarly able to differentiate blood Mo into M2-like MF by co-delivery of colony-stimulating factor (CSF-1) and IL-4 [168]. The delivery system moderately increased the presence of M2-like (CD68^+^CD206^+^) MF near the infarct site and improved cardiac function at day 15. Projahn et al. used thiol-functionalized star-shaped poly(ethylene oxide-stat-propylene oxide) (sP(EO-stat-PO)) and linear poly(glycidol) (PG) degradable hydrogels to temporally control the release of two chemokines: one that reduces neutrophil infiltration (Met-CCL5) within the first few hours and one that stimulates stem cell recruitment (CXCL12 (S4V)) over the course of several weeks post-MI. The delivery of both fast releasing CXCL12 (S4V) and slow releasing Met-CCL5 hydrogels were able to prevent neutrophil migration into the infarcted myocardium, reduce cardiomyocyte apoptosis, and promote vascularization, all of which improved cardiac function after MI [169, 370].

In addition, hydrogels can be an efficacious vehicle for local cell therapy. Typically, the delivery of cell suspensions alone results in poor cell survival, retention, and engraftment (often < 1%) that motivates the need for a biomaterial carrier [371]. Hydrogels can improve cell viability during injection, enhance cell engraftment, and are permeable to allow for oxygen and nutrient diffusion necessary to support continued viability of encapsulated cells in vivo [147, 372]. Liu et al. co-transplanted bone marrow-derived MSCs (BMSCs) with a chitosan hydrogel to increase stem cell retention and modulate the MI immune environment in mice [150]. Application post-MI alleviated the inflammatory response, as reflected by a reduction in TNF-α, IL-6, IL-1β, caspase-11, and caspase-1. It also protected vascular endothelial cells from pyroptosis and attenuated ventricular remodeling. Likewise, Shin et al. encapsulated MSCs in alginate and confined the cells to rat myocardial walls with a PEG hydrogel following MI. The conversion of pro-inflammatory AMP to anti-inflammatory adenosine through MSCs via CD73 reduced initial neutrophil and MF infiltration, prevented ROS formation, and accelerated cardiac repair [148]. It is clear that vehicle-assisted delivery of MSCs holds potential to aid in restoration of cardiac function after MI; though, effects may differ greatly from originally intended direct cardioregeneration.

Incorporating EVs into hydrogels has also become a promising approach, as they can communicate intracellularly and avoid issues often associated with stem cell sourcing and rejection [373]. Lv et al. delivered BMSC-derived EVs from alginate hydrogels [153], finding that the EVs were retained at the infarct site and decreased cardiac fibrosis and cell death, while also promoting M2-like polarization. Similarly, EPC-derived EVs were delivered to the myocardium via shear-thinning guest–host hydrogels, intended to mimic EPC function. Local delivery allowed for sustained release of EPC-derived EVs over 21 days, promoted angiogenesis, and improved ventricular hemodynamic function [154]. In addition to EVs, Zhang et al. revealed the role of dendritic cell-derived exosomes (DEXs) through their delivery in alginate hydrogels [155]. Notably, infiltration of Tregs and M2-like MF was enhanced in the infarct border zone in mice, and DEXs alone were able to induce both Treg and M2-like polarization in vitro. While effects of DEXs alone were short lived, hydrogel-based delivery prolonged the effects and healing capability to create a long-lasting reparatory environment.

Injectable hydrogels are likewise useful as a vehicle for the local delivery of naturally derived or fully synthetic nanoparticles. A recent study used functionalized mesoporous silica nanoparticles (MSNs) instead of EVs to deliver miRNAs, specifically miR-21-5p, thus creating an MSN/miR-21-5p complex [157]. To deliver the MSNs, a pH-responsive injectable hydrogel delivery system was used, which incorporated MSN/miR-21-5p encapsulation into a hydrogel matrix (Gel@MSN/miR-21-5p) that allowed for delivery upon acidic stimulation. The MSNs were able to improve angiogenesis following MI, as well as downregulate TLR2 and subsequently NF-κB signaling, thus decreasing TNF-α, IL-1β, and IL-6 proinflammatory cytokine expression. Yi and colleagues have also reported an injectable filamentous hydrogel for low-dose, sustained delivery of anti-inflammatory nanocarriers. The researchers loaded a bioactive form of vitamin D, which inhibits pro-inflammatory NF-κB, in PEG-b-PPS filomicelles that transition from a cylindrical to spherical morphology to gradually release drug-loaded micelles. After a single subcutaneous treatment in ApoE^−/−^ mice, high levels of regulatory T cells were observed both in atherosclerotic lesions and distant organs for several weeks [173].

In an exciting approach to create cardiac patches in a minimally invasive manner, methods of intrapericardial hydrogel injection have been recently demonstrated for immunotherapeutic delivery. Studies by Ke Cheng and colleagues have encapsulated MSC-derived exosomes or iPSC-derived cardiac progenitor cells in methacrylated HA hydrogels with subsequent injection into the pericardial space, either by direct injection in mice and rats or by a minimally invasive thoracoscope-guided approach in pigs (Fig. 6c) [151, 156]. In mice, hydrogel injection resulted in retention and prolonged release of the exosomes. At endpoint in rats, metrics of LV dilation, fibrosis, and myocyte survival were improved by EV delivery relative to saline and HA controls, while pig studies established safety of the interventional approach.

Throughout these many forms of bulk materials and devices, multiple opportunities arise. It is apparent that these systems confer a unique opportunity as multifunctional interventions, as they can simultaneously target multiple mechanisms of LV remodeling. This includes provision of mechanical restraint, mitigating infarct thinning and expansion to directly address tissue-level remodeling processes. As seen for the CorCap device, mechanical restraint in itself may influence the immune environment post-MI. Direct studies to investigate these interaction affects in detail, however, are largely lacking from the literature. When combined with appropriate materials selection or therapeutics, these local delivery strategies can also directly modulate the hyperinflammatory post-MI milieu that drives continued tissue injury. Importantly, therapeutic delivery is concentrated at the site of action. This is a particularly important consideration in immune modulation, where the systemic administration of immunosuppressive drugs exacerbates the risk of infection or septic shock, which has hindered clinical approval [257, 374]. Conversely, the systemic delivery of immunostimulatory drugs produces a widespread interferon response that likewise mitigates their use. In the case of both immunosuppressive and immunostimulatory strategies, local administration may therefore improve outcomes by targeting action towards the site of injury, prolonging the therapeutic window via controlled release, and by overcoming systemic side effects.

## Conclusion

Immunotherapies are redefining the medical management of disease, enabling the most significant improvements in patient outcomes seen in decades for fields such as cancer treatment and regulation of autoimmune disease. These approaches are now finding their way to regenerative medicine, where cardioimmunology is an emerging frontier open to newfound discoveries in the fundamental pathophysiology of disease development and progression [51, 375]. Emerging evidence reveals that LV remodeling and IHF development, in particular, are driven by both initial and persistent inflammation that continue to damage to the heart. This understanding gives rise to distinct modes of treatment, including cardioprotective and cardioregenerative strategies. Cardioprotective strategies developed to date are largely based on immunosuppression, and are therefore a promising prophylactic approach to abating IHF. On the other hand, leaders in the field have only recently recognized that stimulation of the pro-regenerative immune response is a prime target, and a unique opportunity to reverse deleterious remodeling to restore heart function [376]. Toward each of these general approaches, this review has highlighted interesting counterexamples, wherein pro-inflammatory stimuli (e.g., TLR agonists [11, 212, 273], pro-inflammatory cytokines [166]) are effective in preserving heart function. Overall, these studies are in alignment with the general hypothesis that initial inflammation is essential for subsequent healing, such as by provoking angiogenic response [377, 378]. Though, such functions may be tissue and context-specific [379]. For both immunosuppressive and immunostimulatory therapies in the context of LV remodeling, there remain fundamental gaps in knowledge. As discussed, a more thorough understanding of the therapeutic window is needed, as seen for immunosuppressive treatments through meta-analysis [108]. Moreover, it remains to be seen that cardioregenerative therapies can reverse late-stage remodeling. Related to these considerations, there is a dire need to better characterize the link between the innate and adaptive immune response post-MI. While many studies discussed here have focused on mitigating early inflammation, they fail to characterize downstream effectors, such as B and T cells. This is a crucial consideration, given the demonstrated role of the adaptive immunity in IHF development [33, 38] and the divergent role of regulatory, neonatal, and adult T cells in cardiac regeneration [380, 381].

The continued progress towards immunotherapeutic strategies for IHF is likely to have broader implications for the field. For example, the preservation of donor organs for transplant is essential [382–384]. This is particularly evident in heart transplant, where the acute inflammatory response has been implicated as a driver of tissue injury and waning organ function that ultimately render the organ unusable [385]. Application of appropriate immunosuppressive therapeutics in this context is an interesting strategy to preserve function, whereas regenerative approaches could provide an opportunity to rescue heart function post-transplant. For such therapeutic strategies to become accepted, however, they must first be shown to be safe and effective in clinical use before broader applications may be investigated. Biomaterial-based approaches will continue to aid in this endeavor as they are a platform to provide much needed cell or tissue-targeted delivery that can be multiplexed with the biological function of the material itself, including specific cell-material interactions and the provision of mechanical restraint to attenuate concurrent tissue remodeling.

## Data Availability

Not applicable.
